# M2pep-Modified Cyclodextrin-siRNA Nanoparticles Modulate the Immunosuppressive Tumor Microenvironment for Prostate Cancer Therapy

**DOI:** 10.1021/acs.molpharmaceut.3c00769

**Published:** 2023-10-24

**Authors:** Yao Sun, Michael F. Cronin, Monique C. P. Mendonça, Jianfeng Guo, Caitriona M. O’Driscoll

**Affiliations:** † School of Pharmacy, University College Cork, Cork T12 K8AF, Ireland; ‡ School of Pharmaceutical Sciences, Jilin University, Changchun 130021, China

**Keywords:** targeted nanoparticles, functionalized cyclodextrin, tumor microenvironment, M2pep, M2 macrophages, CSF-1R, prostate cancer, immunotherapy

## Abstract

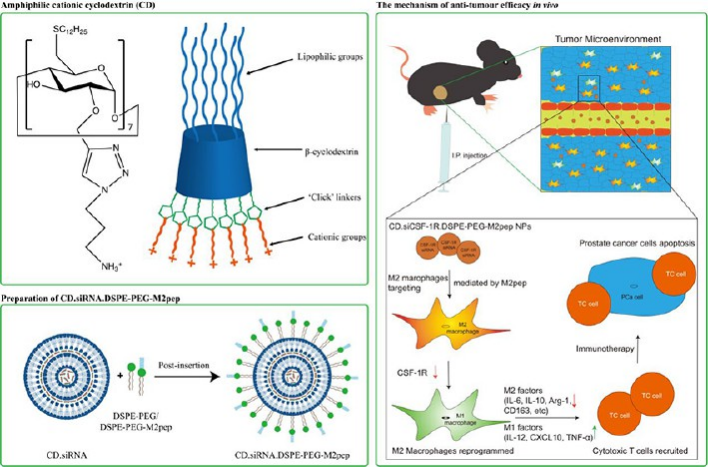

Prostate cancer (PCa) is the most prevalent cause of cancer deaths in men. Conventional strategies, such as surgery, radiation, or chemotherapy, face challenges including poor prognosis and resistance. Therefore, the development of new improved strategies is vital to enhance patient outcomes. Recently, immunotherapy has shown potential in the treatment of a range of cancers, including PCa. Tumor-associated macrophages (TAMs) play an important role in the tumor microenvironment (TME) and reprogramming of TAMs is associated with remodeling the TME. The colony-stimulating factor-1/colony stimulating factor-1 receptor (*CSF-1/CSF-1R*) signaling pathway is closely related to the polarization of TAMs. The downregulation of *CSF-1R*, using small interfering RNA (siRNA), has been shown to achieve the reprogramming of TAMs, from the immunosuppressive M2 phenotype to the immunostimulatory M1 one. To maximize specific cellular delivery an M2 macrophage-targeting peptide, M2pep, was formulated with an amphiphilic cationic β-Cyclodextrin (CD) incorporating *CSF-1R* siRNA. The resulting nanoparticles (NPs) increased M2 macrophage targeting both *in vitro* and *in vivo,* promoting the release of M1 factors and simultaneously downregulating the levels of M2 factors through TAM reprogramming. The subsequent remodeling of the TME resulted in a reduction in tumor growth in a subcutaneous PCa mouse model mainly mediated through the recruitment of cytotoxic T cells. In summary, this M2pep-targeted CD-based delivery system demonstrated significant antitumor efficacy, thus presenting an alternative immunotherapeutic strategy for PCa treatment.

## Introduction

In 2022 in U.S.A. males, approximately 268,490 new cases and about 34,500 deaths from prostate cancer (PCa), estimated to account for 27% of new cancer cases and 11% of deaths, were reported making it the most prevalent and second-deadliest cancer type.^1^ Current treatment options are limited and frequently ineffective; therefore, an urgent need exists to develop more effective clinical alternatives. Recently immunotherapy has shown promise in the treatment of a range of cancers including PCa, which is considered to be an immunosuppressive cancer. TAMs have been regarded as pro-tumoral macrophages and consequently are linked to adverse prognoses in cancers, such as PCa.^2,3^ TAMs and tumor cells are the main cellular components constituting the TME, and cross-talk between these cell types is the important regulator of tumor growth and metastasis.^3^ TAMs are generally classified into two phenotypes, M1 and M2.^4^ M1 TAMs are regarded as an antitumorigenic phenotype, which releases M1 factors, such as interleukin-12 (IL-12) to directly kill tumor cells and recruit cytotoxic T cells for indirect antitumor effects.^5,6^ In contrast, M2 TAMs described as pro-tumoral macrophages are associated with tumor immunosuppression and angiogenesis.^7,8^ The number of M2 TAMs increases with tumor growth and gradually dominates the immunosuppressive tumor microenvironment (TME). The immunosuppressive action of M2 TAMs is due to the release of M2 factors, such as interleukin-6 (IL-6), which can cause resistance to immunotherapeutics.^6,9^ It is known that cytotoxic T lymphocytes (CTLs) are key factors in immunotherapy.^10^ In immunosuppressive cancers such as PCa, the TME suppresses the infiltration of CTLs by multiple mechanisms, including M2 factors released by M2 TAMs.^11^ At the same time, M2 cytokines also upregulate the level of anti-inflammatory cells, such as myeloid-derived suppressor cells (MDSCs) and regulatory T cells (Tregs).^3^ In addition, the subpopulations of MDSCs and M-MDSCs are stimulated by M2 factors to differentiate into M2 macrophages.^12^ This cycle leads to an increasing degree of immunosuppression in the TME. Consequently, modulating the polarization of TAMs from M2 to M1 phenotypes has been reported as a promising immunotherapeutic strategy for cancer.^4−6,9^

The colony-stimulating factor-1 receptor (CSF-1R) plays a critical role in the polarization of TAMs. Downregulation of CSF-1R expression, using small interfering RNA (siRNA) or inhibitors/monoclonal antibodies, has proven to reprogram M2 macrophages to the M1 phenotype.^13,14^ A number of clinical trials including clinic trail Nos.: NCT02323191 (completed, applied anti-CSF-1R, and anti-PD-L1 monoclonal antibodies on solid tumors), NCT02718911 (completed, applied CSF-1R monoclonal antibody on solid tumors), and NCT01346358 (completed, applied CSF-1R monoclonal antibody on solid tumors)] have investigated this approach for cancer therapy.^15−17^

One of the main challenges in manipulating the TME for therapeutic outcomes is the ability to target the cells of interest and avoid nonspecific delivery. Lipid NPs following parenteral administration are preferentially taken up by the liver partially due to the endogenous target mechanism involving binding to Apolipoprotein E.^18^ Consequently, new biomaterials are urgently needed to achieve delivery beyond the liver to other disease targets. Modified cyclodextrins provide an opportunity to achieve a wider level of biodistribution. Cyclodextrins have been used for gene delivery and have shown transfection efficacy in a range of diseases, including cancers and central nervous system (CNS) diseases.^19,20^ In addition, the advantages of cyclodextrins including low toxicity, high transfection efficiency, and the ability to add functional moieties via the hydroxyl groups are major advantages making CDs an attractive option as a nonviral nucleic acid delivery vector.^20,21^ Among these functionalized cyclodextrins, an amphiphilic cationic β-cyclodextrin has promoted siRNA delivery due to its high transfection capacity.^22^ The amphiphilic cationic β-cyclodextrin was substituted on the primary side with lipophilic chains (C12) to improve the transfection efficiency of cationic CDs. Functionalization at the 2 position was by means of a “click” reaction, which represents an efficient and versatile strategy for modifying the secondary side with diverse groups, including cationic groups and PEG chains.^23,24^ Previously, sialic acid-targeted cyclodextrin-based NPs containing siRNA have been studied in PCa immunotherapy, and the results illustrated the ability of the NPs to target the macrophages and remodel the TME.^25^ M2 macrophage-targeting peptide, M2pep, is an artificially designed peptide, the sequence of which was selected by Pun and co-workers using subtractive phage biopanning.^26^ The application of M2pep as a targeting ligand has been investigated previously where polyethylenimine-stearic acid-based micelles with M2pep successfully codelivered CSF-1R siRNA and PI3K-γ inhibitor to reprogram TAMs for pancreatic cancer therapy.^14^ In addition, a dual targeting ligand approach including α-peptide [a scavenger receptor B type 1 (SR-B1) targeting peptide] and M2pep was utilized for the delivery of lipid nanoparticles to treat melanoma.^13^ In the current work, we are investigating a novel delivery approach using M2pep-targeted amphiphilic cyclodextrin (CD)-based NP to incorporate siRNA for prostate cancer treatment. The ability of the resulting CD-siRNA NPs to reprogram the TAM was initially studied *in vitro* and the optimized formulation was subsequently investigated in an *in vivo* PCa model using C57BL/6 mice.

## Materials and Methods

### Materials

1, 2-Distearoyl-*sn*-glycero-3-phosphoethanolamine-poly(ethylene glycol)-2000 (DSPE-PEG) and DSPE-PEG-maleimide (DSPE-PEG2000-Mal) were purchased from Nanocs Inc. (New York, NY, USA). M2pep (sequence: *N*′-Cys-YEQDPWGVKWWY-C’) was synthesized by TAG Copenhagen A/S (Frederiksberg, Denmark). Human GAPDH siRNA (sense sequence: 5′-GUGGAUAUUGUUGCCAUCAtt-3′), mouse GAPDH siRNA (sense sequence: 5′-GGCCGAGAAUGGGAAGCUUtt-3′), human CSF-1R siRNA (sense sequence: 5′-GUUGAGACCUUAGAGCACAtt-3′), and mouse CSF-1R siRNA (sense sequence: 5′-GGUCUUACGCAAAACGGUCtt-3′) were purchased from Thermo Fisher Scientific (Waltham, MA, USA). Negative control siRNA (siNeg), 6-FAM-labeled negative control siRNA (FAM-siNeg), and Cyanine 5-labeled negative control siRNA (Cy5-siNeg) were purchased from Sigma-Aldrich (St. Louis, MO, USA). RNA extraction kits and RNA reverse transcription kits were purchased from Sigma-Aldrich (St. Louis, MO, USA). All primers, RT-PCR kits, and immunofluorescence antibodies were purchased from Thermo Fisher Scientific (Waltham, MA, USA). Flow cytometry antibodies and ELISA kits were purchased from BioLegend (San Diego, CA, USA).

### Cells and Animals

The *Homo sapiens* monocyte cell line THP-1 was donated by Cancer Research @UCC, University College Cork, Ireland. The mouse macrophage cell line RAW 264.7, the *Homo sapiens* prostatic adenocarcinoma bone metastasis cell line PC-3, and the mouse prostatic adenocarcinoma cell line TRAMP-C1 were purchased from the American Type Culture Collection (ATCC, Manassas, VA, USA).

THP-1 cells were cultured in RPMI-1640 medium (Sigma-Aldrich) supplemented with 10% fetal bovine serum (FBS, Sigma-Aldrich), 10 mM HEPES (Sigma-Aldrich), 1 mM sodium pyruvate (Sigma-Aldrich), D-(+)-glucose (Sigma-Aldrich), 0.05 mM beta-mercaptoethanol (Sigma-Aldrich), and 1% penicillin–streptomycin (Sigma-Aldrich). RAW 264.7 cells were cultured in Dulbecco’s Modified Eagle’s Medium (DMEM, Sigma-Aldrich) supplemented with 10% FBS and 1% penicillin–streptomycin. PC-3 cells were cultured in RPMI-1640 medium (Sigma-Aldrich) supplemented with 10% FBS and 1% penicillin–streptomycin. TRAMP-C1 cells were cultured in DMEM supplemented with 5% Nu Serum IV (Corning), 5% FBS, 5 μg/mL insulin (Sigma-Aldrich), 10 nM 5α-androstan-17β-ol-3-one (Sigma-Aldrich), and 1% penicillin–streptomycin.^27,28^ All cells were incubated at 37 °C and supplied with 5% carbon dioxide.

Male C57BL/6 mice (4–6 weeks old) were purchased from Envigo (Bicester, UK). All animals were housed in the Biological Service Unit (BSU), University College Cork, Ireland. The environment is specific pathogen-free and temperature-controlled. All animal experiments were approved by the Animal Experimentation Ethics Committee of University College Cork (AEEC, UCC, No. UCC2021/022) and the Health Products Regulatory Authority (HPRA, No. AE19130/P159). All researchers for animal experiments were well-trained and authorized by HPRA for specific animal experiments.

The monocyte cell line, THP-1, was differentiated to macrophages by treating (48 h) with 200 nM 12-O-tetradecanoylphorbol 13-acetate (PMA, Sigma-Aldrich).^29,30^ Subsequently, 20 ng/mL lipopolysaccharides (LPS, Sigma-Aldrich) or 20 ng/mL recombinant human interleukin-4 (IL-4, PeproTech) was used to differentiate THP-1-derived macrophages to M1 or M2 phenotypes.^31−33^

For RAW 264.7 cells, 20 ng/mL LPS or 20 ng/mL murine IL-4 was used to promote differentiation from the M0 phenotype to M1 or M2 macrophages.^34−36^

### Synthesis of DSPE-PEG-M2pep

M2pep and DSPE-PEG-Mal were conjugated via the Michael addition; the reaction of the cysteine thiol group on M2pep with the DSPE-PEG-Mal maleimide group.^14^ Briefly, DSPE-PEG-Mal (11.70 mg, 0.00585 mmol, 1 equiv) and M2pep (15.44 mg, 0.00877 mmol, 1.5 equiv) were suspended in chloroform/methanol (3 mL, 2/1, v/v). The mixture was sonicated for 30 min to promote dissolution. Then, triethylamine (20 μL) was added as a catalyst. The solution was stirred under a nitrogen atmosphere for 2 days at room temperature. Thin layer chromatography (TLC, silica: chloroform/methanol) comparison of a reaction sample and starting materials, observed under a UV lamp, indicated the complete consumption of DSPE-PEG-Mal. The reaction solution was dialyzed against deionized water (DIW) in a 3.5k Mw cutoff dialysis cassette (Thermo Fisher) for 48 h with outer water replacement every 12 h. The product, DSPE-PEG-M2pep (11.29 mg, 0.003 mmol, 51.33 mol % based on DSPE-PEG-Mal) as a tan-colored solid, was obtained by freeze-drying and stored at −20 °C. DSPE-PEG-M2pep was identified by NMR (Bruker Avance 600 UltraShield NMR spectrometer, USA) and Fourier transform infrared (FT-IR, PerkinElmer Spectrum Two, Attenuated Total Reflectance) spectrometry.

### Nanoparticle Preparation

The amphiphilic cationic β-cyclodextrin (CD) was prepared as previously reported.^37,38^ The CD was dissolved in water and sonicated at 60 °C for 1 h to promote dissolution. For the basic nanoparticles, CD.siRNA, the CD and siRNA aqueous solutions were incubated at 25 °C for 0.5 h at 450 rpm in a ThermoMixer (Eppendorf). The nontargeted and targeted formulations CD.siRNA.DSPE-PEG and CD.siRNA.DSPE-PEG-M2pep were achieved by a “post-insertion” method,^37^ where CD.siRNA complexes were incubated with DSPE-PEG or DSPE-PEG-M2pep at 60 °C for 1 h, at 450 rpm in a ThermoMixer (Eppendorf, Hamburg, Germany).

### Physicochemical Characterization of the Nanoparticles

To optimize the optimum ratio of CD and siRNA, CD.siRNA NPs at different weight ratios (WR) were examined by agarose gel electrophoresis to monitor the binding of siRNA. Free siNeg or CD.siNeg NPs ranging from WR 5:1 to 20:1 were loaded on a 1% (w/v) agarose gel in Tris/Borate/EDTA (TBE) buffer containing a nucleic acid stain (NBS Biologicals Ltd.). The weight of siNeg was fixed at 0.5 μg. Electrophoresis was performed using 120 V for 0.5 h; results were imaged and photographed under UV.

The hydrodynamic size and polydispersity index (PDI) were determined by dynamic light scattering using a Zetasizer NanoZS instrument (Malvern Instruments, Worcestershire, UK), and the zeta potential was measured by laser Doppler microelectrophoresis. CD.siRNA complexes were coformulated with DSPE-PEG in the range of 0.14:1 to 7:1 MR to optimize the ratio of CD and DSPE-PEG. After identification of the optimum WR of CD to siRNA and MR of CD to DSPE-PEG, three formulations of CD.siRNA, CD.siRNA.DSPE-PEG, and CD.siRNA.DSPE-PEG-M2pep were examined by agarose gel electrophoresis and DLS.

The morphology of the different formulations was visualized by transmission electron microscopy (TEM, JEOL 2000 FXII, Jeol Ltd.). CD.siNeg NPs were placed on copper grids for 3 min, stained by uranyl acetate, and imaged after 12 h.

The stability of siRNA in NPs following incubation in 50% FBS for 4 or 24 h was assessed, and the heparin released siRNA was analyzed on an agarose gel. The NPs were incubated with salt-containing media (90% Opti-MEM media, Thermo Fisher) for up to 3 days, and sizes were measured by DLS every 24 h.

### *In Vitro* Cellular Uptake in M2 Macrophages

THP-1 and RAW 264.7 cells were differentiated to M2 macrophages, as described above, and identified by flow cytometry. Briefly, postdifferentiation cells were stained with human (CD68) or mouse (F4/80) and relative M1 (CD86) or M2 (CD206) macrophage markers and the expression of markers was measured by flow cytometry. For THP-1-derived macrophages, CD68^+^/CD86^+^ cells were regarded as human M1 macrophages, and CD68^+^/CD206^+^ cells were regarded as human M2 macrophages. For RAW 264.7-derived macrophages, F4/80^+^/CD86^+^ cells were regarded as mouse M1 macrophages, and F4/80^+^/CD206^+^ cells were regarded as mouse M2 macrophages.

The cellular uptake of targeted and nontargeted NPs containing FAM-siNeg was monitored by flow cytometry and fluorescence microscopy. 1 × 10^5^ M2 macrophages were seeded in 24-well plates, and either targeted or nontargeted NPs were added onto the cells for 6 or 24 h; post-transfected cells were analyzed by flow cytometry or stained by a nuclear acid dye for fluorescence microscopy.

To confirm the targeting effects of NPs, a competitive uptake study was performed, where free M2pep was added to the cells for 1 h; subsequently, the cells were incubated with either targeted or nontargeted NPs for 6 h. After incubation, the cells were analyzed by flow cytometry.

To verify the specificity of the ligand, GAPDH gene knockdown by either targeted or nontargeted NPs containing GAPDH siRNA in both THP-1- and RAW 264.7-derived M0/M1/M2 macrophages was quantified. All macrophage phenotypes were differentiated as described above and seeded on 24-well plates; cells were incubated with the CD formulations for 6 h; the medium was then replaced by the fresh medium for a further 42 h. Postincubation, cells were collected, and the expression of GAPDH was measured by RT-qPCR, and beta-actin was used as the internal reference.

### *In Vitro* Cytotoxicity of Nanoparticles

To examine the cytotoxicity of the three CD formulations, 1 × 10^4^ M2 macrophages were seeded in a 96-well plate, all 3 CD NPs were added to cells for 2 days, the Cell courting kit-8 (CCK-8) solution was added, and the viability was determined using a plate reader (Spark, Tecan, Switzerland) at 460 nm. The cell viability was calculated using the following equation where the untreated group was the negative control and regarded as 100% cell viability.
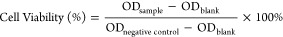


### *In Vitro* Downregulation of CSF-1R

THP-1- and RAW 264.7-derived M2 macrophages were seeded in 24-well plates, different formulations, including Free siRNA, CD.siRNA, CD.siRNA.DSPE-PEG, CD.siRNA.DSPE-PEG-M2pep containing CSF-1R siRNA, and CD.siRNA.DSPE-PEG-M2pep containing siNeg were incubated with the cells for 6 h, and the media was then replaced by complete media for a further 42 h. The expression of CSF-1R was analyzed by RT-qPCR, using beta-actin as the internal reference.

### *In Vitro* Reprogramming of TAMs and Remodeling of the TME

To examine the reprogramming of the M2 macrophages, post-transfected cells were stained using the appropriate antibodies, human (CD68) or mouse (F4/80), and the relevant M1 (CD86) or M2 (CD206) macrophage markers were measured by flow cytometry.

In addition, to further explore the influence of reprogramming of TAMs on immunotherapy, a Transwell model (3 μm pore size, 662630, Greiner Bio-One, Stonehouse, U.K.) was established to simulate the tumor microenvironment by coculturing macrophages and prostate cancer cells. 5 × 10^4^ M2 macrophages were seeded into the upper chamber (100 μL) of the Transwell model, and 1 × 10^5^ PCa cells were seeded on the lower well (600 μL). THP-1-derived M2 macrophages were cocultured with PC-3 cells, and RAW 264.7-derived M2 macrophages were cocultured with TRAMP-C1 cells. Different formulations containing 100 nM siRNA were added into upper chambers for 48 h; post-transfection, the media from the lower wells were collected, and M1/M2 cytokines IL-12/IL-6 protein content was measured by ELISA. Total RNA from the upper chambers was extracted to measure M1 (IL-12, CXCL-10, TNF-α) and M2 (IL-6, IL-10, Arg-1, CD163) factor expression by PT-qPCR. The apoptosis of cancer cells was analyzed by propidium iodide (PI)/Annexin V double staining and measured by flow cytometry.

### *In Vivo* Tumor Accumulation of NPs

A TRAMP-C1 prostate carcinoma graft model was established by subcutaneous injection of 5 × 10^6^ cells into 6-week-old male C57BL/6 mice. Animals were divided into 3 groups (*n* = 3), including a free siRNA group, nontargeted, and targeted NPs groups. The body weight and tumor size were recorded every second day, and tumor volume was calculated as follows:
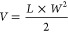


*V* = Volume of the tumor (mm^3^)

*L* = Longest dimension of the tumor (mm)

*W* = Shortest dimension of the tumor (mm)

When the tumor volume reached 100 mm^3^, the animals were treated with the different formulations containing Cy5-siNeg by intraperitoneal (i.p.) injection, the dose of siRNA was 1 mg/kg. At 1, 2, 4, 6, and 24 h post injection, the animals were anesthetized by isoflurane and imaged using an IVIS Spectrum system (IVIS Lumina, PerkinElmer, Waltham, MA, USA). Before imaging, the hair on the tumor sites was shaved carefully. Finally, the animals were euthanatized by cervical dislocation, organs including the heart, liver, spleen, and kidneys were collected and imaged using the IVIS Spectrum system to quantify ex vivo the biodistribution of the NPs in the major organs.

### *In Vivo* Antitumor Effects

The TRAMP-C1 prostate carcinoma model was built as described above. Thirty mice were grouped into 6 groups randomly (*n* = 5). When the tumor volume reached 100 mm^3^, the mice were treated with different formulations, and the dose of siRNA was 1 mg/kg. Animals were treated by i.p. injection every second day on 5 occasions. At 10 days post final treatment, the animals were sacrificed by cervical dislocation. Major organs, including the heart, liver, spleen, lung, kidneys, and tumors, were collected and fixed in 10% formalin at 4 °C for 2 days. Then, all tissues were transferred into 70% ethanol (v/v) and embedded in paraffin. All formalin-fixed paraffin-embedded (FFPE) tissue blocks were cut into 5 μm thickness sections by a rotary microtome (HISTOCORE BIOCUT, Leica Biosystems, Wetzlar, Germany). Hematoxylin and eosin staining (H&E staining) was performed on organs and tumor sections, and terminal deoxynucleotidyl transferase dUTP nick-end labeling (TUNEL staining) was performed on tumor sections. The apoptosis area was brown after TUNEL staining by 3,3′-Diaminobenzidine tetrahydrochloride (DAB), and the apoptosis rate of each group was determined by ImageJ.

### *In Vivo* Infiltration of T Cells

The infiltration of T cells into tumor tissues was measured by immunofluorescence staining assay as follows. Tumor sections were incubated with the T cell marker FITC-antimouse CD3 antibody for 12 h at 4 °C, and the nucleus was stained by DAPI and imaged by fluorescence microscopy. To identify CTLs and T helper cells, CD3 (T cell marker), CD8 (CTL marker), or CD4 (T helper cell marker) expression was measured by flow cytometry. Briefly, the tumor tissue was cut into small pieces and homogenized. Red blood cells were lysed, and the cell suspension was stained with APC-CD3/PE-CD8 antibodies or APC-CD3/FITC-CD4 antibodies for 30 min on ice, avoiding light, and measured by flow cytometry (*n* = 3). To determine the infiltration of regulatory T cells (Tregs), CD3^+^/CD4^+^ T helper cells were separated and then stained with a PE-CD25 (Treg marker) antibody to measure the proportion of CD3^+^/CD4^+^/CD25^+^ cells by flow cytometry (*n* = 3).^39^

### *In Vivo* Reprogramming of Tumor-Associated Macrophages

To determine the *in vivo* reprogramming of TAMs, the tumor tissue was cut into small pieces and homogenized, after lysis of red cells, the total RNA of cells was extracted, and CSF-1R and M1 (IL-12, CXCL10, TNF-α)/M2 (IL-6, IL-10, Arg-1, CD163) factor expression in tumor tissue was measured by RT-qPCR, and M1 (IL-12)/M2 (IL-6) cytokine expression was measured in the cell suspension by ELISA. The expression of M1 (CD86) and M2 markers (CD206) in tumor sections was determined by immunofluorescence staining. Sections were incubated with PE-antimouse CD86 and FITC-antimouse CD206 antibodies for 12 h at 4 °C and the nucleus stained by DAPI was imaged by fluorescence microscopy. To measure the proportion of M1 and M2 macrophages, cell suspension was stained with mouse macrophage/M1 cell markers APC-F4/80/PE-CD86 or mouse macrophage/M2 cell markers APC-F4/80/FITC-CD206 antibodies and measured by flow cytometry (*n* = 3).

### Statistical Analysis

The statistical analysis was performed with Prism 8.0 (GraphPad, San Diego, CA, USA). Data were shown as mean ± standard deviation (mean ± SD) from at least 3 independent experiments. One-way analysis of variance (one-way ANOVA), followed by Dunnet or Tukey post hoc tests and Student’s *t*-test was used to calculate the statistical significance between three or more groups and two groups. A *p*-value < 0.05 was considered a significant difference.

## Results and Discussion

### Preparation and *In Vitro* Characterization of the Nanoparticles

The M2pep targeting moiety was successfully conjugated to DSPE-PEG via the Michael Reaction.^14^ The structure of the product DSPE-PEG-M2pep was confirmed by ^1^H/^13^C NMR and FT-IR spectroscopy (Figure S1).

**Figure 1:**
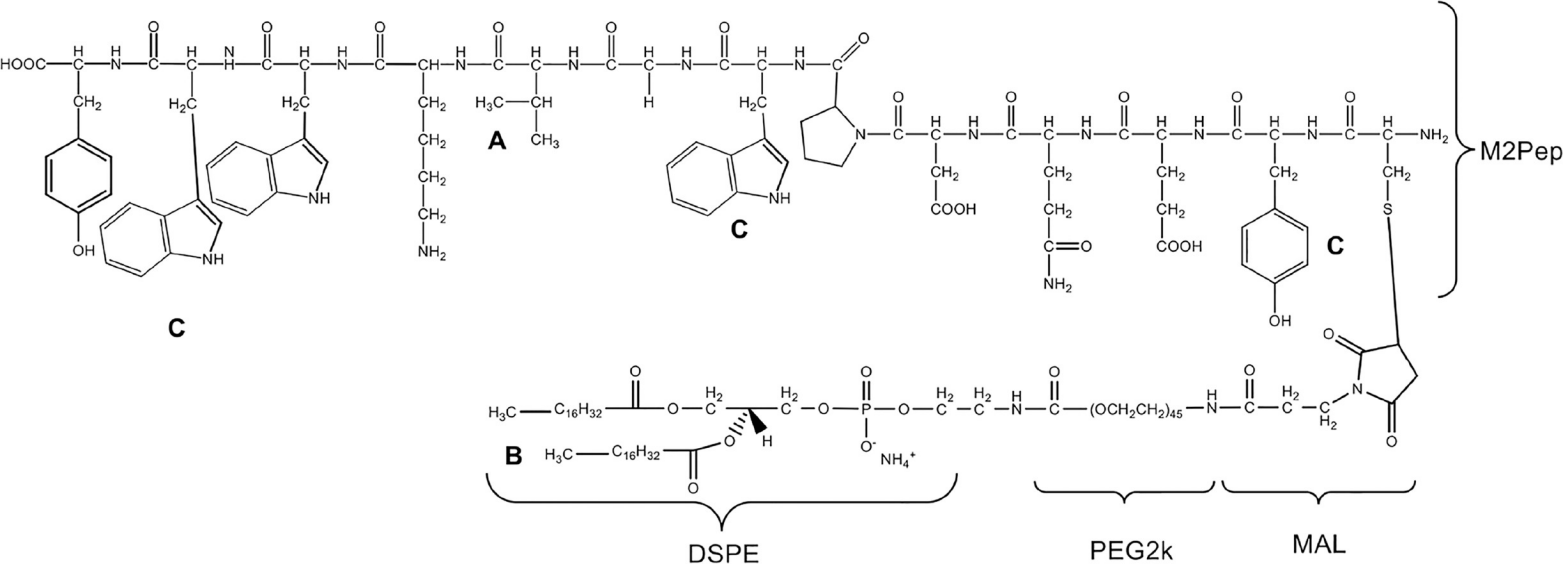
Chemical structure of the ligand, DSPE-PEG-M2pep.

Key signals associated with M2pep are evident in the proton spectrum: tryptophan and tyrosine aromatic residues (marker C in Figures 1 and S1) in the 6.6–8.5 ppm region. Methyl group protons associated with valine and also terminal methyl groups of DSPE alkyl chains (markers A and B respectively in Figures 1 and S1) are observed at 0.7–0.9 ppm. A strong PEG2k signal at 3.3–3.5 ppm and C17 alkyl chain protons resonating in the 1.0–1.9 ppm area are diagnostic for DSPE-PEG. The carbon spectrum has corresponding aromatic signals in the 110–130 ppm region (tryptophan and tyrosine), PEG2k carbons (−OCH_2_−) at 70 ppm, and alkyl chain carbons in the range 20–35 ppm.

The FT-IR spectrum of the conjugate is shown in Figure S1 in comparison to M2pep and DSPE-PEG-Mal starting materials. Diagnostic signals observed for the conjugate are amide I (1633 cm^–1^) and amide II (1547 cm^–1^), both associated with M2pep, along with PEG (C–O–C stretch) (1094 cm^–1^).

^1^H NMR (DMSO, ppm): δ 0.7–0.9 (m, 12H), 1.1–1.9 (m, 24H), 2.0–2.1 (m, 4H), 2.2–2.3 (m, 3H), 2.6–3.9 (m, 192H), 4.05 (s, 1H), 4.1–4.6 (m, 10H), 4.82 (s, 1H), 4.98 (s, 1H), 5.10 (s, 1H), 6.60 (m, 4H), 6.75 (s, 1H), 6.9–7.4 (m, 15H), 7.50 (m, 2H), 7.60 (m, 1H), 7.7–8.5 (m, 11H), 9.20 (br s, 1H), 10.8 (m, 2H).

^13^C NMR (DMSO, ppm): δ 19.63, 22.45, 26.84, 27.35, 28.14, 28.63, 29.00, 29.28, 29.84, 30.08, 31.80, 35.85, 36.06, 36.57, 39.82, 39.88, 39.96, 40.03, 40.06, 40.09, 40.20, 40.23, 40.31, 40.34, 40.48, 40.86, 44.09, 46.15, 46.72, 47.31, 52.49, 53.87, 54.56, 60.66, 63.51, 69.55, 70.02, 70.15, 70.18, 70.25, 72.79, 73.11, 74.48, 75.92, 102.22, 111.70, 115.40, 118.67, 118.76, 121.25, 121.34, 124.01, 124.09, 130.58.

FT-IR cm^–1^: 3385 (O–H), 2956, 2920, 2850 (C–H), 1633 (amide I), 1547 (amide II), 1094 (C–O–C).

Initially, weight ratios (WR) of CD to siRNA in a range of WR 5:1 to WR 20:1 were formulated; in all cases, full binding (agarose gel electrophoresis) of siRNA was achieved (Figure S2A). Particle size and surface charge were measured by DLS (Figure S2B). Optimum results were detected as follows with WR 10:1, CD to siRNA: size 171.1 ± 20.6 nm, charge 13.2 ± 2.6 mV, and PDI of 0.23 ± 0.02. Therefore, CD.siRNA WR 10:1 was used for all further studies (Table 1).

**Table 1:** Size and Charge of the Optimized CD Formulations^a^

formulation	size (nm)	PDI	charge (mV)
CD.siRNA	171.1 ± 20.6	0.23 ± 0.02	13.2 ± 2.6
CD.siRNA.DSPE-PEG	189.6 ± 29.1	0.35 ± 0.01	14.2 ± 2.7
CD.siRNA.DSPE-PEG-M2pep	252.5 ± 16.5	0.25 ± 0.02	10.8 ± 2.5

a PDI = polydispersity index; mean ± SD, *n* = 6.

The CD.siRNA NPs were PEGylated by the insertion of DSPE-PEG.^37,40^ Optimization studies identified the following CD.siRNA.DSPE-PEG (WR of CD to siRNA = 10, MR of CD to DSPE-PEG = 0.35:1) (Figure S3): size 189.6 ± 29.1 nm, charge 14.2 ± 2.7 mV, and PDI of 0.35 ± 0.01.

**Figure 2:**
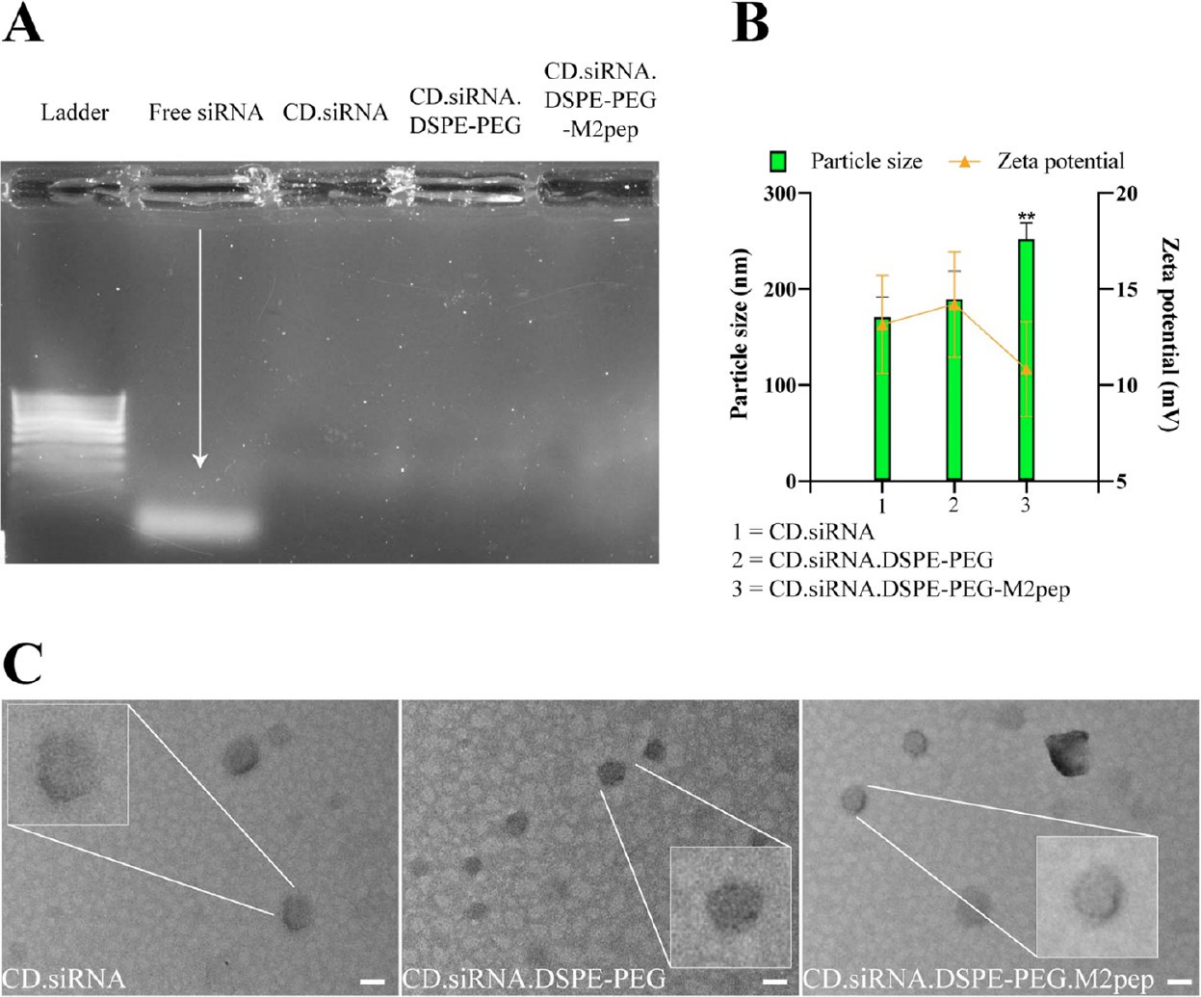
(A) CD formulations containing 0.5 μg of siRNA were confirmed by 1% agarose gel electrophoresis and photographed by UV. (B) Particle sizes and zeta potentials of CD NPs (*n* = 6, mean ± SD, **p < 0.01 compared with CD.siRNA.DSPE-PEG NPs). (C) Morphology of CD formulations (scale bar = 200 nm).

Following incorporation of the targeting moiety DSPE-PEG-M2pep, full binding of siRNA was maintained (Figure 2A). The targeted NPs, CD.siRNA.DSPE-PEG-M2pep, had a size of 252.5 ± 16.5 nm and a charge of 10.8 ± 2.5 mV (Figure 2B and Table 1). The increasing size compared with the nontargeted NPs suggests the successful incorporation of M2pep (*n* = 6, **p < 0.01).

The morphology of the NPs was imaged by TEM (Figure 2C). All particles are spherical, the presence of PEG appears to give a slight halo effect, and sizes were consistent with the DLS results and our previously published CD NPs data.^37,41^

The stability of all three formulations was assessed following incubation with 50% serum. The heparin release data indicate that siRNA is stable following formulation into the NPs (Figure S4A). In addition, relative to naked siRNA, the targeted NPs maintained siRNA stability following incubation in serum for up to 4 and 24 h (Figure S4B). These results were consistent with previous stability reports for similar CD NPs obtained following exposure to human blood.^37^ In addition, stability in 90% Opti-MEM medium (salt-containing) for 3 days was enhanced by the presence of PEG in the targeted and PEGylated nontargeted NPs, thus implying the potential for longer circulation *in vivo* (Figure S4C).

### Cellular Uptake into M2 Macrophages

**Figure 3:**
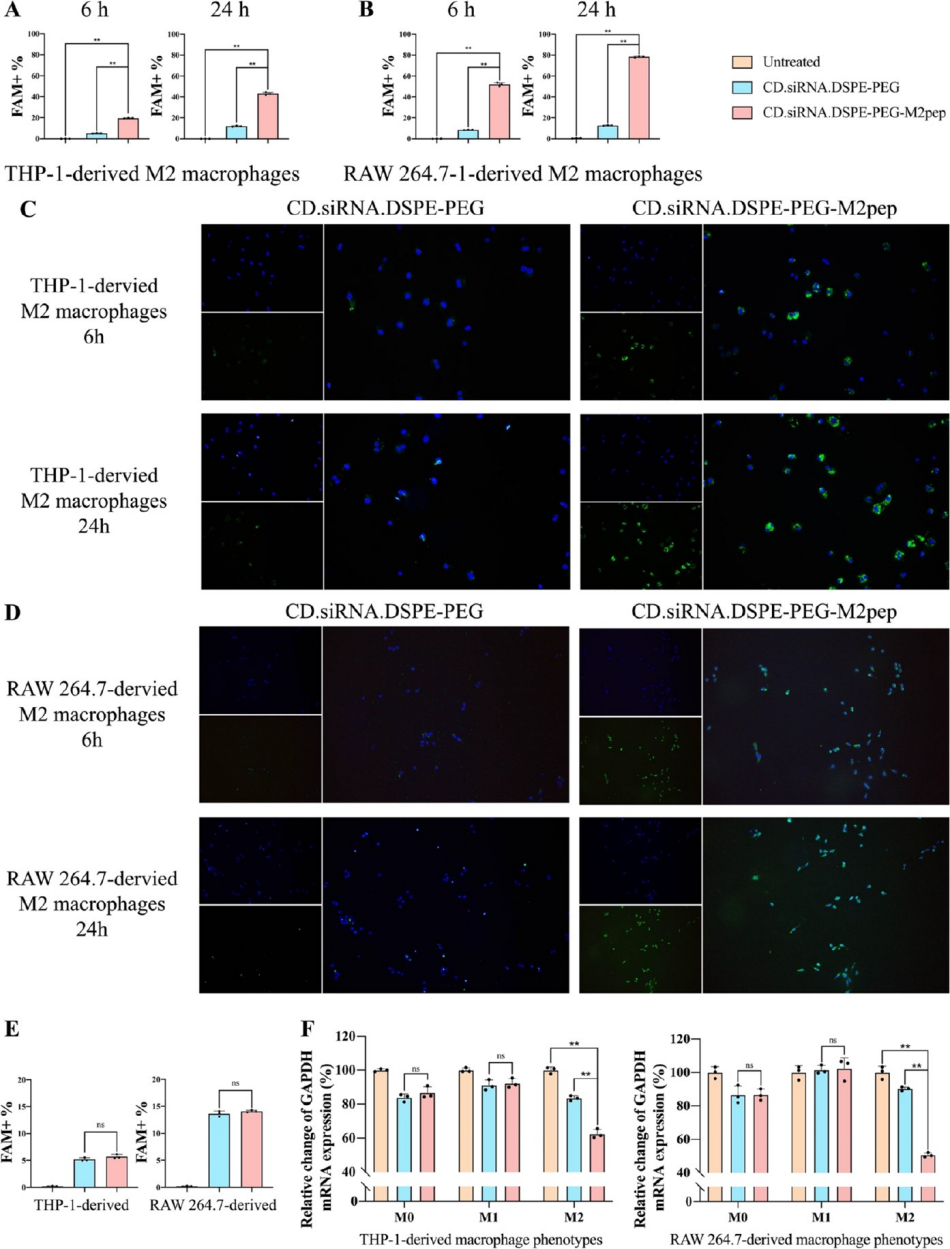
Cellular uptake of CD.siRNA.DSPE-PEG or CD.siRNA.DSPE-PEG-M2pep formulations containing FAM-siRNA in (A) THP-1- or (B) RAW 264.7-derived M2 macrophages after 6 or 24 h transfection (*n* = 3, mean ± SD, **p < 0.01). (C, D) Cellular uptake imaged by fluorescence microscopy (blue: DAPI, green: FAM-siRNA). (E) Competitive uptake, free M2pep was added to THP-1- or RAW 264.7-derived M2 macrophages prior to the addition of the formulations. The CD.siRNA.DSPE-PEG or CD.siRNA.DSPE-PEG-M2pep nanoparticles containing FAM-siRNA were incubated with the cells for 6 h (*n* = 3, ns = not significant). (F) M2 macrophage-targeting efficacy (gene silencing) using CD formulations with GAPDH siRNA in THP-1- or RAW 264.7-derived macrophages, cells were incubated with CD complexes for 6 h followed by replacement with complete media for a further 44 h incubation, analysis by RT-qPCR (*n* = 4, ns = not significant, mean ± SD, **p < 0.01).

THP-1 and RAW 264.7 were differentiated into M2 macrophages as described above, identified by flow cytometry to measure the expression of macrophage markers (Figure S5), and used to investigate cellular uptake. In THP-1-derived M2 macrophages, uptake was higher with the targeted NPs versus the nontargeted (Figures S6A and 3A, 19.49 ± 0.29% FAM^+^ vs 5.22 ± 0.31% FAM^+^ after 6 h, and 43.10 ± 1.45% FAM^+^ vs 11.97 ± 0.60% FAM^+^ after 24 h, *n* = 3, **p < 0.01). Similar results were observed in RAW 264.7-derived M2 macrophages (Figures S6B and 3B), 52.00 ± 1.81% FAM^+^ vs 8.46 ± 0.19% FAM^+^ after 6 h, and 78.40 ± 1.45% FAM^+^ vs 12.67 ± 0.43% FAM^+^ after 24 h (*n* = 3, **p < 0.01). These results indicate that the incorporation of the targeting ligand, M2pep, enhanced the uptake of the NPs. The higher uptake of the targeted NPs after 6 and 24 h was confirmed by fluorescent microscopy (Figure 3C,D). It has been reported that M2pep-targeting formulations increased M2 macrophage cellular internalization by 4.2-fold versus nontargeted NPs.^14^ In this study, a similar increase was observed; after the longer transfection time (24 h), the M2pep-targeting NPs enhanced the levels of cellular internalization by human and mouse M2 macrophages up to 3.6- and 6.2-fold, respectively, compared with the nontargeted CD.siRNA.DSPE-PEG NPs.

To confirm that the higher uptake was due to the targeting ligand, a competitive uptake study was performed. Following exposure of the cells to free M2pep for 1 h, subsequently, no difference in uptake of the targeted versus the nontargeted NPs was detected (*p* = 0.1447 in THP-1-derived M2 macrophages; *p* = 0.2898 in RAW 264.7-derived M2 macrophages) (Figures S6C and 3E). These results indicate the key role of M2pep in the enhanced cellular uptake of the targeted NP.

To verify the gene silencing efficacy of the targeted NPs, we used GAPDH siRNA was used. Post transfection (6 h), no gene knockdown was detected in M0 or M1 macrophages, in contrast to the M2 macrophages, expression of *GAPDH* mRNA was significantly decreased by the targeted versus the nontargeted NPs (Figure 3F, 62.45 ± 2.68% vs 83.42 ± 1.52% in THP-1-derived M2 macrophages, and 50.62 ± 1.35% vs 90.26 ± 1.09% in RAW 264.7-derived M2 macrophages, *n* = 3, **p < 0.01). These results indicate that M2pep labeled NPs are capable of cell specific delivery mainly to M2 macrophages. Compared with sialic acid-targeted CD-based NPs, the M2pep-targeting ligand produced a higher cellular uptake in M2 macrophages (1.13- and 1.24-fold higher in THP-1- and RAW 264.7-derived M2 macrophages after 24 h transfection), indicating the stronger M2 macrophage targeting ability of M2pep.^25^

### *In Vitro* Macrophage Reprogramming

Initially the cytotoxicity of the CD formulations was assessed in both THP-1 and RAW 264.7-derived macrophages using a CCK-8 kit; results show no significant toxicity (*n* = 3, *p* > 0.05), under the conditions studied (Figure S7). While the positive control group, RNAiMAX presented significant cytotoxicity on both THP-1- and RAW 264.7-derived M2 macrophages (*n* = 3, **p < 0.01).

**Figure 4:**
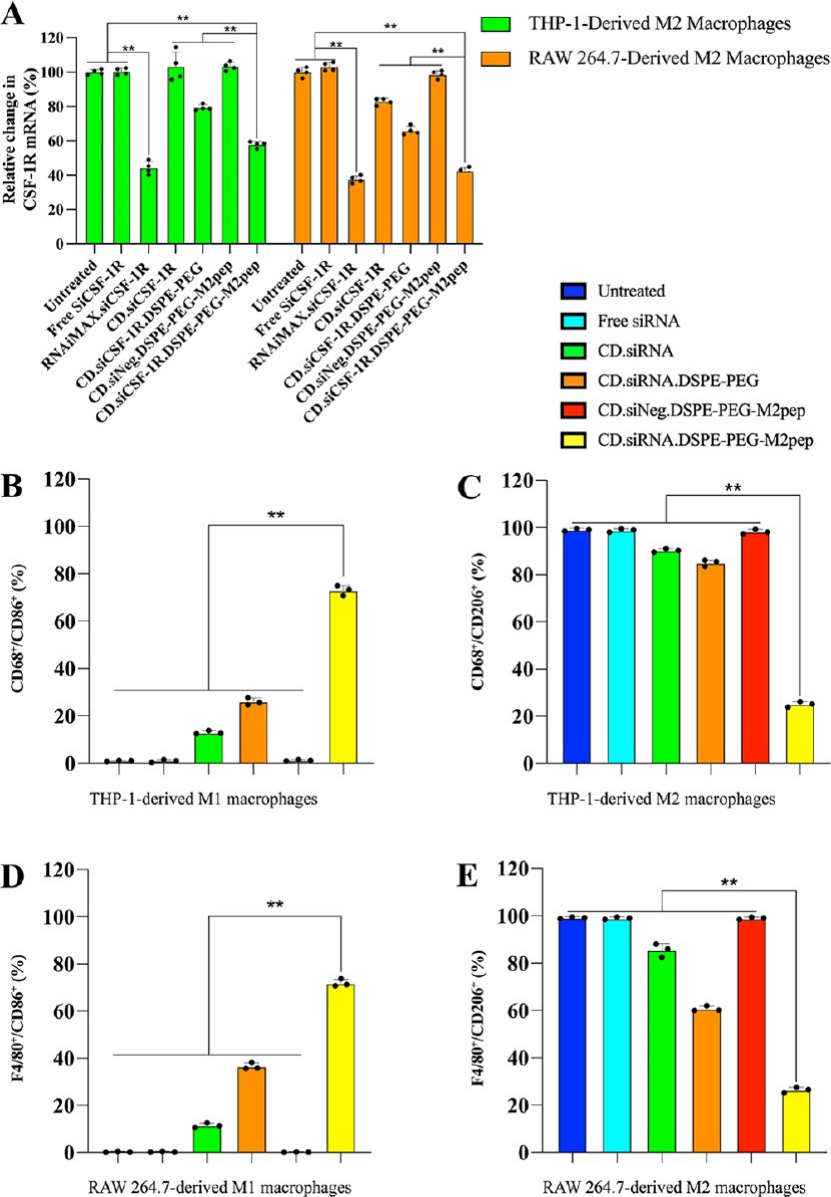
(A) *In vitro* downregulation of *CSF-1R* mRNA, formulations were incubated with THP-1- or RAW 264.7-derived M2 macrophages for 6 h followed by replacement with complete media for a further 44 h incubation (*n* = 4, **p < 0.01). (B–E) Percentage of human/mouse macrophages markers CD68/F4/80 and M1/M2 markers CD86/CD206 expression in THP-1- or RAW 264.7-derived macrophages after transfection by different formulations (*n* = 3, mean ± SD, **p < 0.01).

The ability of the CD NPs to silence *CSF-1R* was assessed in both THP-1- or RAW 264.7-derived M2 macrophages (Figure 4A). The targeted NPs significantly downregulate (*n* = 4, **p < 0.01) the expression levels of *CSF-1R* mRNA in both human (42.18% knockdown) and mouse M2 macrophages (57.65% knockdown). The decrease in gene expression, relative to controls, detected with the PEGylated nontargeted nanoparticles (*n* = 4, **p < 0.01) may be indicative of enhanced stability due to the presence of PEG. In addition, compared with the positive control group, RNAiMAX.siRNA, the targeted NPs presented similar *CSF-1R* mRNA downregulation in RAW 264.7-derived M2 macrophages (*p* = 0.0968). As previously reported, NPs containing CSF-1R siRNA modified with M2pep-targeting ligand downregulated up to 85% expression of *CSF-1R* in pancreatic cancer therapy *in vitro*.^14^ In addition, a dual-targeting ligand [M2pep and α-peptide (an SR-B1 targeting peptide)] for the treatment of melanoma resulted in 81% gene knockdown *in vitro*.^13^

To verify that downregulation of *CSF-1R* achieved the reprogramming of TAMs from the M2 to the M1 phenotype, specific macrophage markers were measured in the post-transfected cells (Figure S8A,B). Relative to untreated controls, in the THP-1-derived macrophages, the targeted NPs significantly increased the M1 macrophages (CD68^+^/CD86^+^) to 72.97 ± 0.21% and decreased the M2 macrophages (CD68^+^/CD206^+^) to 25.03 ± 1.10% (Figure 4B,C, *n* = 3, **p < 0.01). Similar results were observed in mouse macrophages (Figure S8C,D), where the M1 macrophages (F4/80^+^/CD86^+^) were increased to 71.90 ± 1.57% and the M2 macrophages (F4/80^+^/CD206^+^) were decreased to 26.37 ± 1.21% following exposure to the targeted NPs (Figure 4D,E, *n* = 3, **p < 0.01). The data show that the downregulation of CSF-1R resulted in an increased expression of the M1 markers, indicating the successful reprogramming of the TAM to the M1 phenotype.

Compared with sialic acid-targeted NPs containing CSF-1R siRNA, the M2pep-based formulation presented higher CSF-1R gene silencing efficiencies (increased by 1.43- and 1.48-fold in THP-1- and RAW 264.7-derived M2 macrophages, respectively) and TAMs reprogramming rates (increased by 1.35- and 1.07-fold in THP-1- and RAW 264.7-derived M2 macrophages, respectively), the higher transfection efficiencies are mainly due to the higher cellular uptake mediated via M2pep.^25^

### *In Vitro* Antitumor Effects

The macrophage reprogramming for killing tumor cells was monitored using a Transwell coculture model, where THP-1- or RAW 264.7-derived M2 macrophages were seeded into the upper chamber, and human prostate cancer PC-3 cells or mouse prostate cancer TRAMP-C1 cells were seeded into the lower chamber.

**Figure 5:**
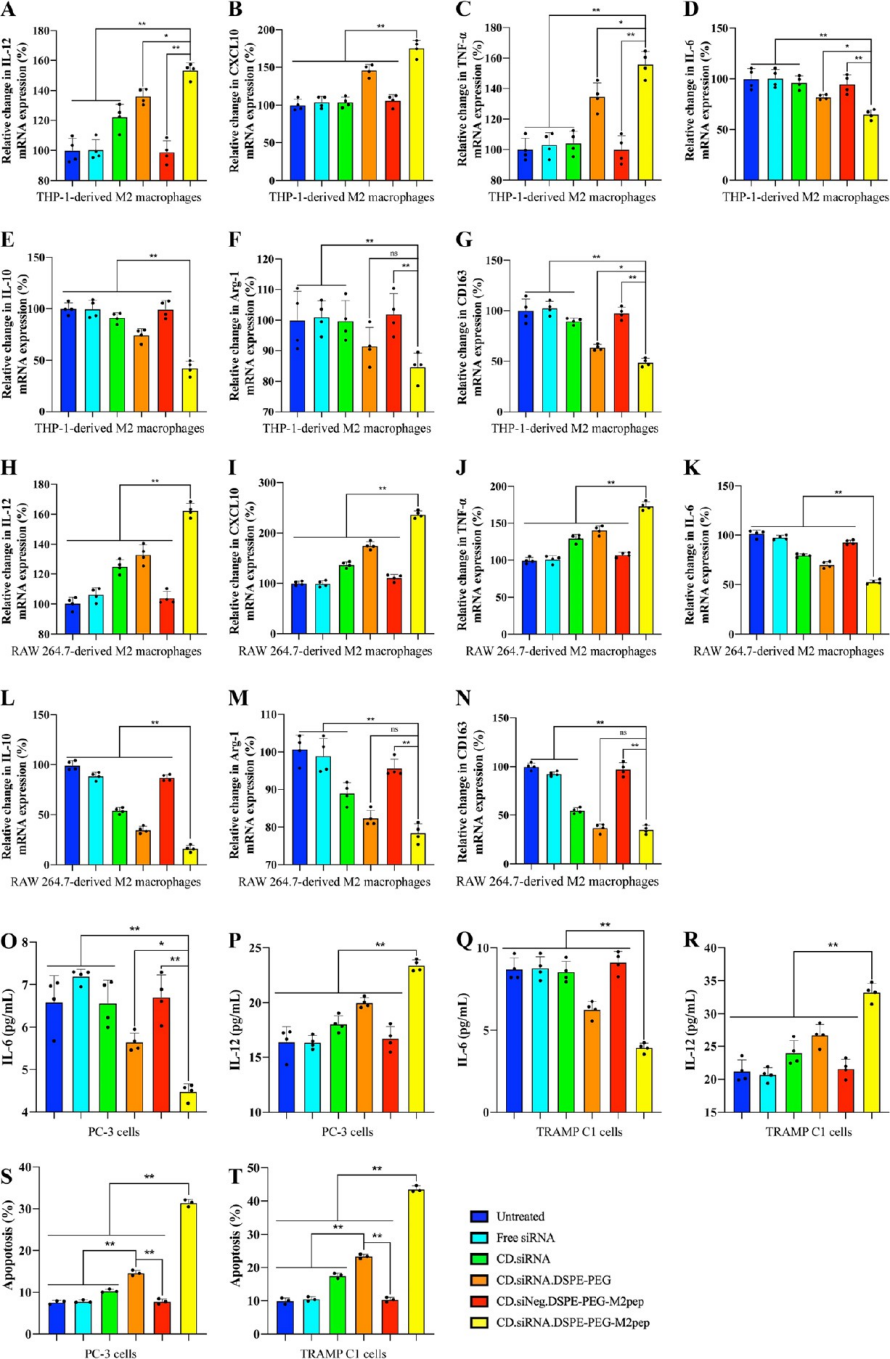
Coculture of M2 macrophages and prostate cancer cells. M2 macrophages were seeded in the upper chambers of the Transwell model, and prostate cancer cells were seeded in the lower chambers. RT-qPCR results of M1 cytokines in THP-1-derived macrophages: (A) IL-12, (B) CXCL-10, and (C) TNF-α; M2 cytokines in THP-1-derived macrophages: (D) IL-6, (E) IL-10, (F) Arg-1, and (G) CD163. M1 cytokines in RAW 264.7-derived macrophages: (H) IL-12, (I) CXCL-10, and (J) TNF-α,and M2 cytokines in RAW 264.7-derived macrophages: (K) IL-6, (L) IL-10, (M) Arg-1, and (N) CD163. Elisa results of cytokines in PC-3 cells: (O) IL-6, and (P) IL-12, and in TRAMP-C1 cells: (Q) IL-6, and (R) IL-12 (*n* = 4, ns = not significant, mean ± SD, *p < 0.05, **p < 0.01). Percentage of apoptosis of (S) PC-3 and (T) TRAMP-C1 cells in the lower chambers of the Transwell model (*n* = 3, mean ± SD, **p < 0.01).

Following exposure to the NPs, expression of the M1/M2 factors in the macrophages was measured using RT-qPCR (Figure 5A–N), and expression of M1/M2 cytokines in the supernatant of the lower chamber was quantified by ELISA (Figure 5O–R). Results show that the targeted NPs significantly increased the expression of M1 factors, including IL-12, CXCL10, and TNF-α and decreased the expression of M2 factors, including IL-6, IL-10, Arg-1, and CD163 in both human and mouse macrophages (*n* = 3, *p < 0.05, **p < 0.01). The ELISA results (Figure 5O–R) confirmed the upregulation of IL-12 and the downregulation of IL-6 (*n* = 3, *p < 0.05, **p < 0.01).

Apoptosis of cancer cells was measured by PI/Annexin V double staining and flow cytometry. Flow cytometry results are shown in Figure S9A,B, and the percentage of apoptosis is shown in Figure 5S,T. Compared with the other groups, the targeted nanoparticles increased cancer cell apoptosis in both PC-3 cells (31.37 ± 0.83%) and TRAMP-C1 (43.55 ± 1.01%) (*n* = 3, **p < 0.01). The increase in apoptosis, relative to controls, detected with the nontargeted nanoparticles (14.61 ± 0.63% in PC-3 cells; and 23.36 ± 0.65% in TRAMP-C1, *n* = 3, **p < 0.01) may be indicative of enhanced stability and subsequently enhanced transfection due to the presence of PEG. It has been previously reported that polyethylenimine–stearic acid-based NPs containing CSF-1R siRNA and the M2pep-targeting ligand achieved approximately 40% apoptosis of cancer cells in a Transwell coculture model of M2 macrophages and pancreatic cancer Pan-02 cell lines.^14^

In summary, the *in vitro* results indicate that NPs containing siRNA delivered using the CD-based biomaterial successfully reprogrammed the TME, promoting an immunostimulatory response, and the degree of apoptosis reported here for prostate cancer compares well with the previously reported efficacy data in pancreatic cancer.^14^

### *In Vivo* and *Ex Vivo* Biodistribution of M2pep Targeted NPs

**Figure 6:**
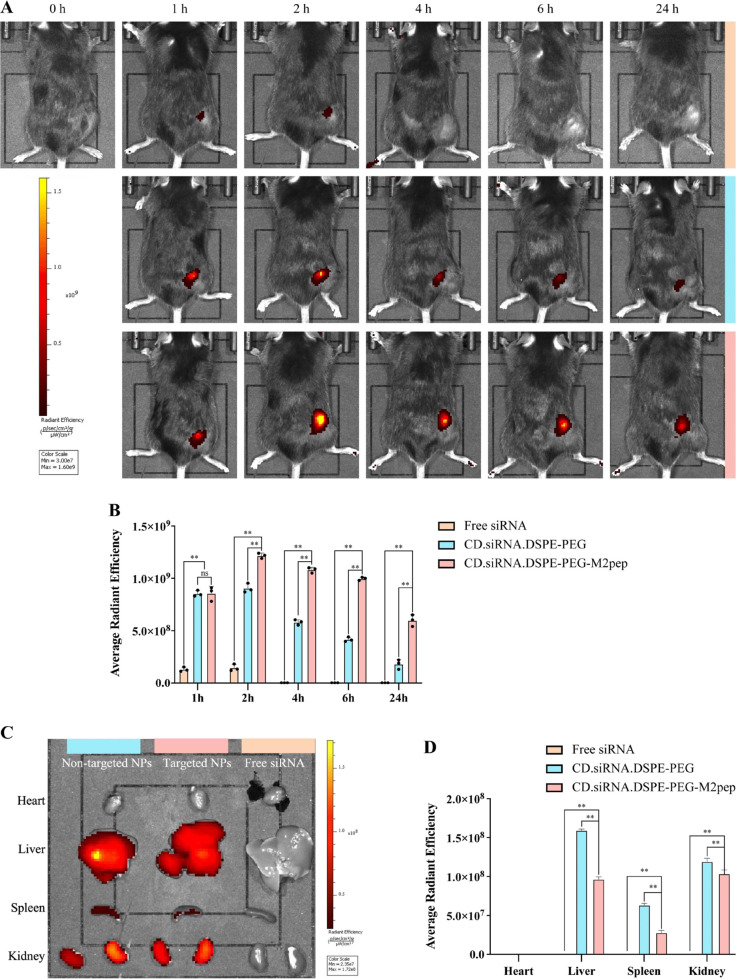
(A) *In vivo* tumor accumulation of (i) free siRNA (top line, orange); (ii) CD.siRNA.DSPE-PEG (middle line, blue); and (iii) CD.siRNA.DSPE-PEG-M2pep (bottom line, pink) labeled with Cy5-siRNA at 1 h, 2 h, 4 h, 6 h, and 24 h after injection. (B) Semiquantitative analysis of tumor accumulation (*n* = 3, ns = not significant, mean ± SD, **p < 0.01). (C) Ex vivo biodistribution of Cy5-siRNA 24 h postinjection in organs of different formulations. (D) Semiquantitative analysis of tissue biodistribution (*n* = 3, mean ± SD, **p < 0.01).

A time course study indicated that tumor accumulation of NPs labeled with Cy5-siRNA was maximized at 2 h post injection (Figure 6A). With the free siRNA group (Figure 6A), only a weak signal was detected at 1 and 2 h, with no signal monitored at other time points. In comparison, the targeted nanoparticles showed a significantly higher tumor accumulation of Cy5-siRNA relative to other groups (1.35-fold versus nontargeted at peak levels, up to 3.32-fold at 12 h postadministration, Figure 5B, **p < 0.01). The animals were euthanized after final IVIS imaging; the major organs were collected, and ex vivo accumulation of Cy5-siRNA was monitored. Free siRNA was not detected in any organ. In contrast to the targeted NPs, the nontargeted NPs appeared to accumulate in the liver and kidneys (Figure 6C,D), this is consistent with the lower Cy5-siRNA accumulation detected previously in tumors for the nontargeted NPs (**p < 0.01).

The results indicate that the formulation of siRNA within NPs increased the *in vivo* stability and avoided rapid degradation in the systemic circulation relative to naked siRNA, this is consistent with our *in vitro* stability results. Targeted NPs accumulated in the tumor area; in contrast, nontargeted NPs displayed higher accumulation in the liver and kidneys, indicating the targeting efficiency of the M2pep ligand. When the same ligand was used to target pancreatic tumors (intravenous injection), a similar level (∼2.4-fold versus nontargeted NPs at peak levels) of accumulation in the tumor tissue was detected, albeit the time (4 h) to reach peak levels was later.^14^

### *In Vivo* Antitumor Effects

**Figure 7:**
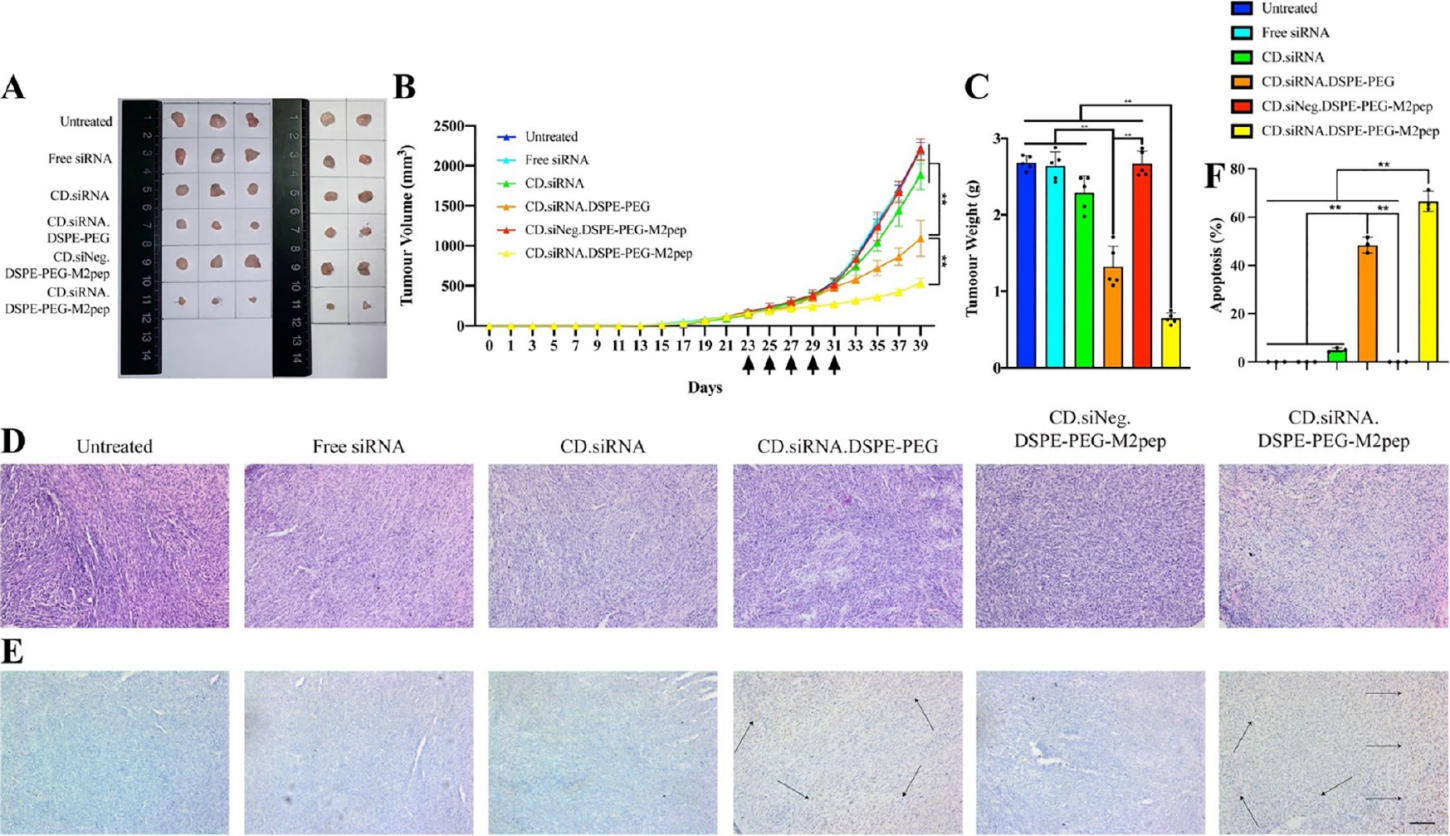
(A) Images of tumor tissues from the prostate cancer-bearing models on day 39. (B) Tumor volumes over the course of the study following i.p administration on D23, D25, D27, D29, D31 (black arrows) of NPs with siRNA against CSF-1R (D39: *n* = 5, mean ± SD, **p < 0.01). (C) Tumor weight from the prostate cancer-bearing models on day 39 (*n* = 5, mean ± SD, **p < 0.01). (D) H&E staining of tumor sections for controls and NP treatments. (E) TUNEL staining of tumor sections for controls and NP treatments (black arrow: apoptosis areas), hematoxylin was used for counterstaining the nucleus. Scale bar = 100 μm. (F) Level (%) of apoptosis in the tumor sections (*n* = 3, mean ± SD, **p < 0.01).

The *in vivo* antitumor effects were analyzed in a TRAMP-C1 prostate carcinoma mouse model following i.p. administration. Following treatment with the targeted NPs containing siRNA against CSF-1R, both tumor volumes (Figure 7A,B) and weights (Figure 7C) significantly decreased (75.88% in tumor size decrease and 75.90% in tumor weight decrease, compared with the untreated group **p < 0.01), compared with other groups. During the study, the body weight of tumor-bearing animals increased gradually in all cases, suggesting no toxicity due to the formulations administered (Figure S10).

On day 39, the animals were euthanatized, H&E staining was performed on the tumor (Figure 7D) and organ sections (Figure S11), and TUNEL staining was performed on tumor sections (Figure 7E). The tumor section staining results indicated that the targeted NPs promoted higher levels of apoptosis relative to controls (Figure 7F, **p < 0.01), which is consistent with the tumor volumes and weights results. Examination of the slides from the major organs did not show any evidence of toxicity.

In summary, the CD-based M2pep-targeted CSF-1R siRNA NPs achieved a significant reduction in tumor volumes (75.88%) and tumor weights (75.90%) compared with untreated groups. These results compare well with previously published data, where 1,2-dimyristoyl-*sn*-glycero3-phosphocholine (DMPC)- and cholesterol oleate (C.O)-based M2pep/α-peptide-dual targeted NPs containing CSF-1R siRNA reduced the tumor size by 87% in melanoma-bearing animal models.^13^ In addition, a tumor size reduction of (73%) was observed in an M2pep-targeted polyethylenimine-stearic acid–based NPs with CSF-1R siRNA and PI3K-γ inhibitor in a pancreatic cancer model, while the NPs containing only CSF-1R siRNA achieved around a 50% reduction in tumor size.^14^

### *In Vivo* Remodeling of the Tumor Microenvironment

**Figure 8:**
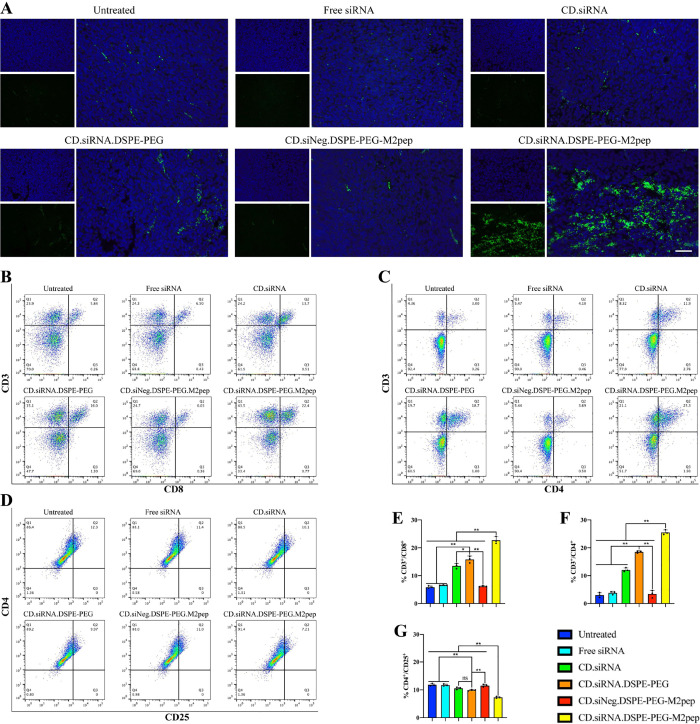
(A) Immunofluorescence of T cells infiltration in tumor sections. Green: FITC-CD3. Blue: DAPI. Scale bar = 100 μm. (B) Cytotoxic T cells (CD3^+^/CD8^+^) infiltration in tumor tissues. (C) T helper cells (CD3^+^/CD4^+^) infiltration in tumor tissues. (D) Regulatory T cells (CD3^+^/CD4^+^/CD25^+^) infiltration in tumor tissues. (E) Percentage of cytotoxic T cells (CD3^+^/CD8^+^) infiltration (*n* = 3, mean ± SD, *p < 0.05, **p < 0.01). (F) Percentage of T helper cells (CD3^+^/CD4^+^) infiltration (*n* = 3, mean ± SD, **p < 0.01). (G) percentage of Regulatory T cells (CD3^+^/CD4^+^/CD25^+^) infiltration in tumor tissues (*n* = 3, ns = not significant, mean ± SD, **p < 0.01).

To investigate the mechanism of the antitumor effects and the remodeling of the TME, T cell infiltration in the tumors was monitored. Following immunofluorescence staining a higher T cell level was observed after treatment with the targeted NPs (Figure 8A). Different types of T cells were separated by flow cytometry; CTLs were CD3^+^/CD8^+^ (Figure 8B), and T helper cells (Th cells) were CD3^+^/CD4^+^ (Figure 8C). The results indicated that the targeted NPs recruited significantly more T cells in the tumor area relative to other treatment groups (Figure 8E–F, **p < 0.01). The upregulation of T cell infiltration is a key sign of TME remodeling; the targeted NPs increased CD8^+^ CTLs 3.8-fold versus the untreated group. A similar trend has been reported where M2pep-targeted polyethylenimine–stearic acid-based NPs simultaneously containing CSF-1R siRNA and NVP-BEZ 235 (a PI3K-γ inhibitor) increased the levels of CD8^+^ CTLs 1.5-fold compared with the untreated group in a pancreatic cancer animal model, while the M2pep-targeted NP containing CSF-1R siRNA produced a 1.1-fold increasing in the CD8^+^ CTL level.^14^ In a further study, NPs formulated with DMPC and C.O containing CSF-1R siRNA with an M2pep and α-peptide dual ligand achieve a 2.7-fold higher CD8^+^ CTL level in a melanoma-bearing mouse model.^13^

The proportion of regulatory T cells (Tregs) within Th cells was monitored by flow cytometry using CD25 as a Treg marker for detection in the CD3^+^/CD4^+^ populations. The results show that the proportion of Tregs following treatment with the targeted NPs was significantly lower than the other groups (Figure 8D,G) (**p < 0.01), suggesting that the targeted formulation downregulated the immunosuppressive T cells in the tumor while the higher infiltration of CD8^+^ CTLs marked the successfully remodeled TME. This trend is consistent with the antitumor effects described above.

### *In Vivo* Reprogramming of TAMs

**Figure 9:**
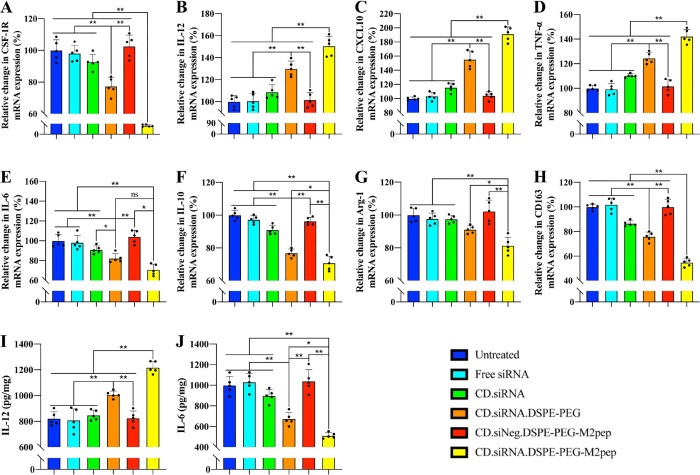
(A) *In vivo* downregulation of CSF-1R by formulations containing CSF-1R siRNA. (B–D) Upregulation of M1 factors by CD formulations at the gene level, including IL-12, CXCL10, and TNF-α. (E–H) Downregulation of M2 factors by CD formulations at the gene level, including IL-6, IL-10, Arg-1, and CD163. (I, J) Protein levels of IL-12 and IL-6 (*n* = 5, ns = not significant, *p < 0.05, **p < 0.01).

As the upregulation of immunostimulatory cytokines like IL-12 can promote CTL activation, RT-qPCR and ELISA were performed to measure both M1 and M2 factor expression in tumor samples. As the data above indicate, TME remodeling and T cell infiltration play significant roles in promoting the antitumor effects, as both are caused by the reprogramming of TAMs, and the latter was subsequently measured. Treatment with the targeted NPs silenced the expression of CSF-1R at the mRNA level (Figure 9A), resulting in significant downregulation of M2 factors (Figure 9E–H) and upregulation of M1 factors (Figure 9B–D) relative to other treatment groups (*p < 0.05, **p < 0.01). These results were confirmed at the protein expression level, as shown by ELISA (Figure 9I, J).

**Figure 10:**
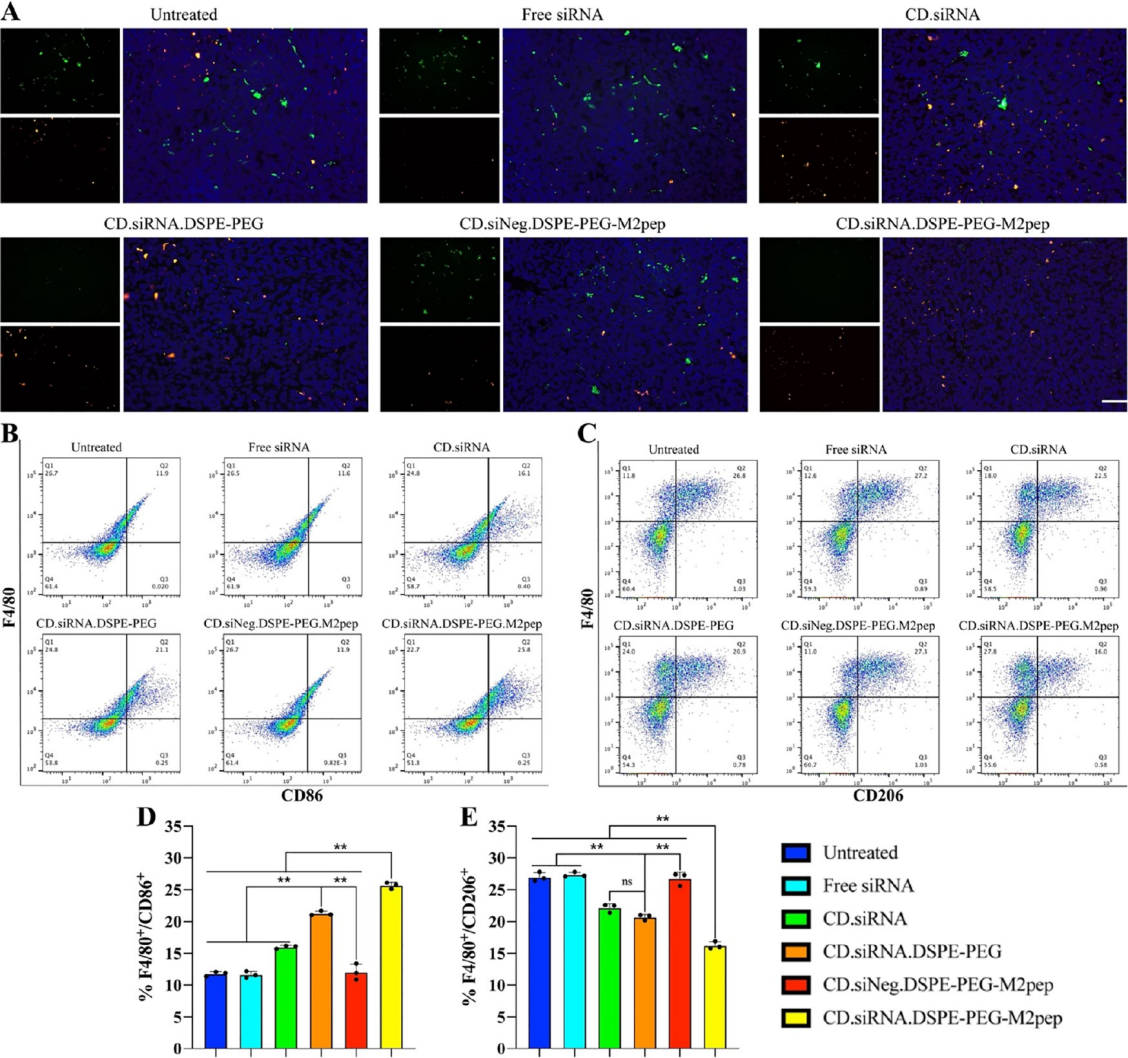
(A) Immunofluorescence of the reprogramming of TAMs. Blue: DAPI, green: FITC-CD206, and red: PE-CD86. Scale bar = 100 nm. (B) M1 macrophages (F4/80^+^/CD86^+^) level in tumor tissues. (C) M2 macrophages (F4/80^+^/CD206^+^) level in tumor tissues. (D) Percentage of M1 macrophages (F4/80^+^/CD86^+^) and (E) Percentage of M2 macrophages (F4/80^+^/CD206^+^). (*n* = 3, ns = not significant, *p < 0.05, **p < 0.01).

In addition, the expression of M1/M2 macrophage markers was tested via immunofluorescence and flow cytometry to analyze TAMs phenotypes. The M1 marker, CD86, and the M2 marker, CD206 in tumor sections, were monitored by immunofluorescence staining (Figure 10A). Results suggested that the M1 marker was higher in the targeted nanoparticle group with a lower M2 marker level. This trend was confirmed by flow cytometry measuring the proportion of M1 macrophages (F4/80^+^/CD86^+^) and M2 macrophages (F4/80^+^/CD206^+^) (Figure 10B,C). More M2 macrophages were reprogrammed to the M1 phenotype following treatment with the targeted nanoparticles compared to the other treatment groups (**p < 0.01) (Figure 10D,E).

In summary, as PCa is an immunosuppressive cancer, an immunotherapeutic approach provides an alternative strategy to design more clinically effective treatments. The CSF-1/CSF-1R pathway has been reported as a potential target for cancer immunotherapy. Downregulation of CSF-1R by inhibitors, antibodies or siRNA has been shown to limit tumor growth in a variety of tumor models.^42,43^ The repolarization of TAMs mediated this tumor limitation by silencing the CSF-1/CSF-1R pathway. The repolarized M1 macrophages release M1 factors including cytokines (IL-12, TNF-α, etc.) and chemokines (CXCL10, etc.) to recruit CD4^+^/CD8^+^ cytotoxic T cells to the tumor area to directly kill tumor cells. At the same time, downregulation of the M2 macrophage level leads to a reduction in the recruitment of immunosuppressive cells, such as MDSCs and Tregs. The higher level of immunostimulatory cell recruitment and the lower level of immunosuppressive cell recruitment causes the remodeling of TME which subsequently achieves the PCa immunotherapy.^9,12,44,45^

To enable clinical translation of siRNA, an effective and safe delivery system is essential. In the current study, a CD-based NP was designed for CSF-1R siRNA delivery with an M2pep-targeting ligand chosen to specifically target M2 macrophages. Both *in vitro* and *in vivo* data show the targeted NPs achieved CSF-1 gene knockdown, resulting in cell apoptosis in the PCa cell models and inhibition of tumor growth in the animal model. Mechanistic studies indicated that the antitumor efficacy was due to high T cell infiltration, including TC cells, and Th cells, promotion of higher levels of M1 macrophages, and a simultaneous decrease in M2 macrophage levels.

The immunotherapy approach, exploited in this work, is based on remodeling the TME landscape by reprogramming M2 macrophages to the M1 ones. Functionalized CDs have previously been used successfully for siRNA delivery in cancer gene therapy.^28,46^ The flexibility of the CD ring structure facilitates the covalent attachment of various functional groups, such as cationic groups to bind the negatively charged siRNA. In addition, the attachment of the lipid component results in a self-assembling amphiphilic CD which can simultaneously increase membrane permeability and facilitate the coformulation, via post insertion, with other lipid entities, in this case, the DSPE-PEG-M2pep targeting ligand. The resulting CD-based targeted nanoparticles have shown efficacy in the *in vivo* PCa mouse model by successfully remodeling the TME.

In addition to the formulation approach used in this work, CDs as biomaterials have the added bonus of providing an opportunity to incorporate small lipophilic anticancer drugs within the hydrophobic cavity, by inclusion complex formation, thus facilitating a combination therapy approach using the same delivery system with the potential for stronger antitumor efficacy.^47,48^

## Conclusions

In the current study, a smart drug delivery system was constructed to deliver CSF-1R siRNA, and the system was based on an amphiphilic cationic CD and an M2 macrophage-targeting ligand DSPE-PEG-M2pep, incorporated into the delivery system via a “post-insertion” method. The specific targeting efficacy for M2 macrophages was verified *in vitro* and *in vivo*. In addition, the NPs reprogrammed M2 macrophages by silencing CSF-1R mRNA expression; in turn, the reprogrammed TAMs remodeled the TME, reactivating the infiltration of CD4^+^/CD8^+^ T cells while decreasing the Tregs level, culminating in PCa cells apoptosis in a PCa bearing mouse model. While the application of gene therapy-based immunotherapy in PCa has previously been explored *in vitro*,^25^ to our knowledge, this is the first report describing *in vivo* efficacy, thus illustrating the potential to translate this strategy for prostate cancer therapy.

## Supporting Information Available

NMR spectra and FT-IR spectra of the ligand DSPE-PEG-M2pep; particle sizes and zeta potentials of CD.siRNA formulations at various weight ratios of CD to siRNA; particle sizes and zeta potentials of CD.siRNA.DSPE-PEG formulations at various molar ratios of CD to DSPE-PEG; stability of CD-based nanoparticles in serum- and salt-containing media; representative flow cytometry figures of M1 and M2 macrophage differentiation; representative flow cytometry figures of cellular uptake; cytotoxicity of CD-based nanoparticles on M2 macrophages; representative flow cytometry figures of M2 macrophages reprogrammed; representative flow cytometry figures of prostate cancer cell apoptosis; body weight changes of tumor-bearing models during the *in vivo* antitumor study; and H&E staining of major organs (PDF)
