# Supplementary material for: M2pep-Modified Cyclodextrin-siRNA Nanoparticles Modulate the Immunosuppressive Tumor Microenvironment for Prostate Cancer Therapy
Source: Mol Pharm. 2023 Oct 24;20(11):5921–36. doi: 10.1021/acs.molpharmaceut.3c00769 (PMC10630955; doi:10.1021/acs.molpharmaceut.3c00769)
Supplement: Supplementary file 1 — mp3c00769_si_001.pdf [file mp3c00769_si_001.pdf]

M2pep-Modified Cyclodextrin-siRNA Nanoparticles Modulate the Immunosuppressive Tumour Microenvironment for Prostate Cancer Therapy

Yao Sun[a], Michael F. Cronin[a], Monique C. P. Mendonça[a], Jianfeng Guo[b*], and Caitriona M. O'Driscoll[a*]

[a] School of Pharmacy, University College Cork, Cork T12 K8AF, Ireland

[b] School of Pharmaceutical Sciences, Jilin University, Changchun 130021, China

*Email: caitriona.odriscoll@ucc.ie (C. M. O'Driscoll), jguo@jlu.edu.cn (J. Guo)

Supporting Information

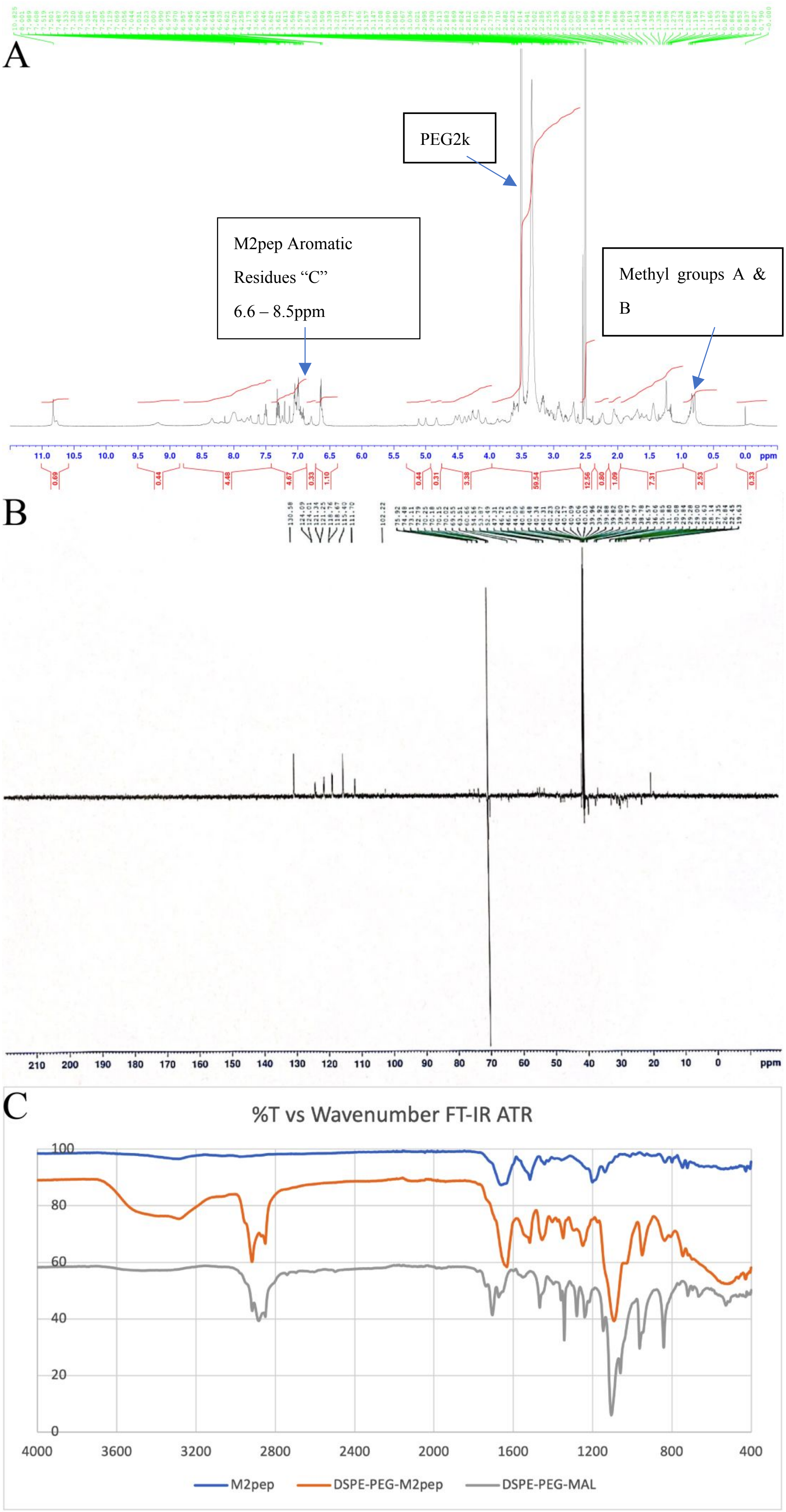
A
PEG2k
M2pep Aromatic
Residues "C"
6.6 – 8.5ppm
Methyl groups A &
B
11.0 10.5 10.0 9.5 9.0 8.5 8.0 7.5 7.0 6.5 6.0 5.5 5.0 4.5 4.0 3.5 3.0 2.5 2.0 1.5 1.0 0.5 0.0 ppm
0.69 0.44 4.48 4.67 0.33 1.10 0.44 0.31 3.38 59.54 12.56 0.80 1.09 7.31 2.53 0.33
B
210 200 190 180 170 160 150 140 130 120 110 100 90 80 70 60 50 40 30 20 10 0 ppm
C
%T vs Wavenumber FT-IR ATR
100
80
60
40
20
0
4000 3600 3200 2800 2400 2000 1600 1200 800 400
M2pep
DSPE-PEG-M2pep
DSPE-PEG-MAL

Figure S1. (A) ^{1}H NMR spectrum of DSPE-PEG-M2pep; (B) ^{13}C NMR spectrum of DSPE-PEG-M2pep, and (C) FT-IR spectra of M2pep, DSPE-PEG-Mal, and DSPE-PEG-M2pep.

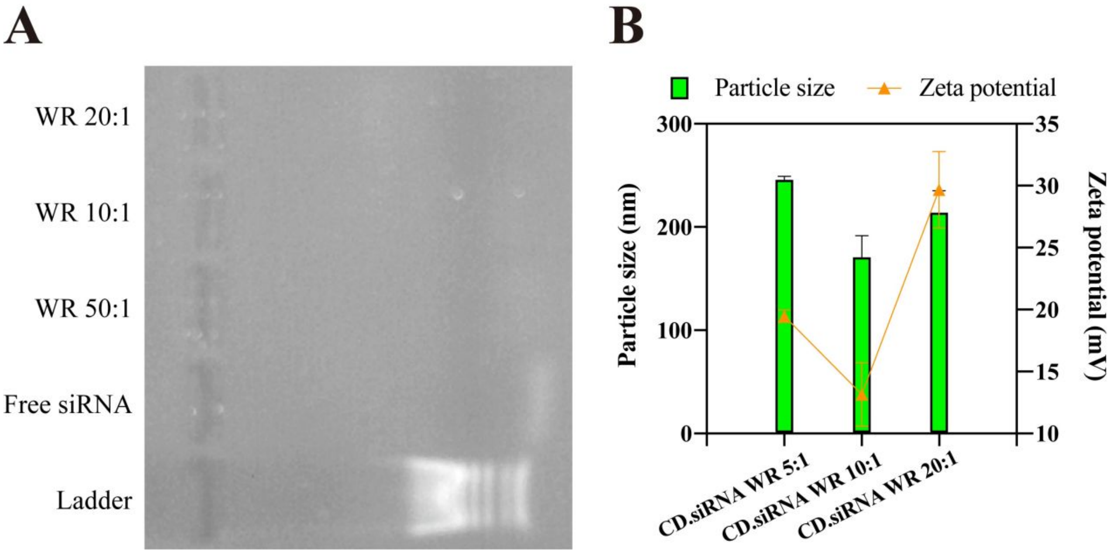

Figure S2. Physicochemical characterization of CD.siRNA formulation at weight ratios ranging of CD to siRNA from 5:1 to 20:1. (a) The complexation of siRNA (0.5 μg) with CD was confirmed by using 1% agarose gel electrophoresis, the resulting gels were imaged under UV. (b) Particle sizes and zeta potentials of CD.siRNA nanoparticles (n = 6, mean ± SD) were measured by DLS.

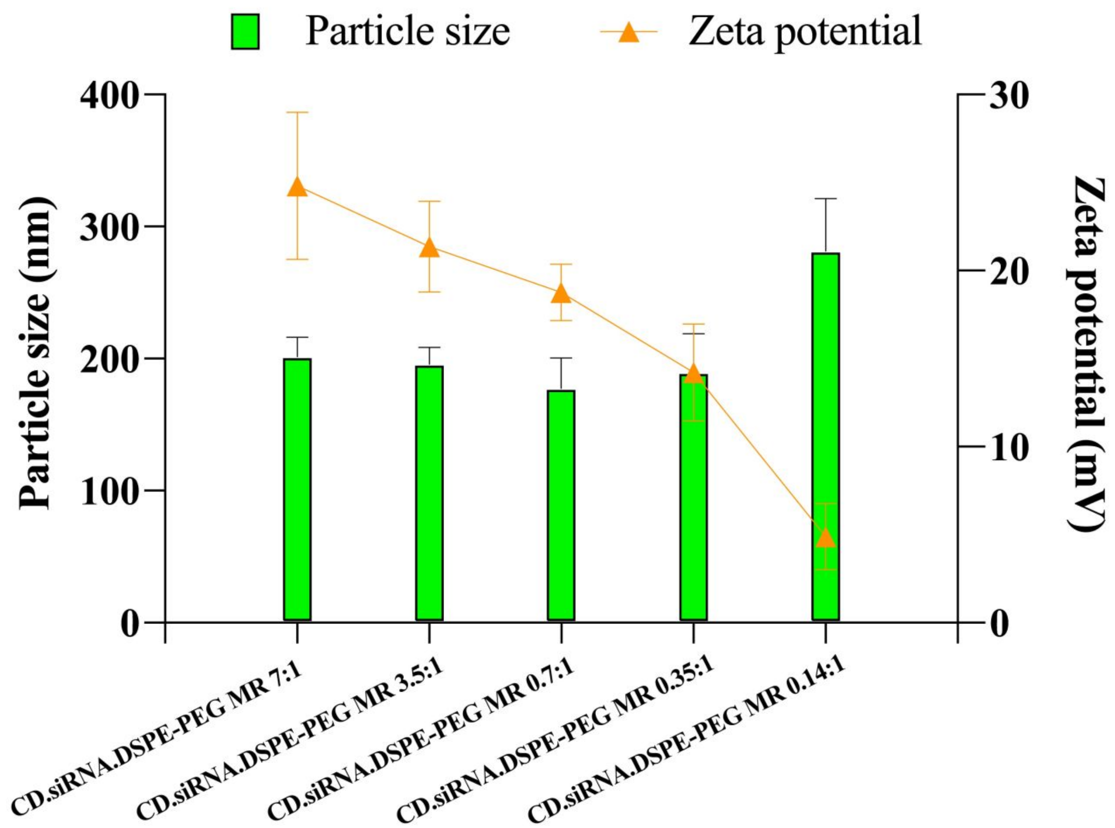

Figure S3. Particle sizes and zeta potentials of CD.siRNA.DSPE-PEG nanoparticles (n = 6, mean ± SD) at molar ratios ranging of CD to DSPE-PEG from 0.14:1 to 7:1 were measured by DLS.

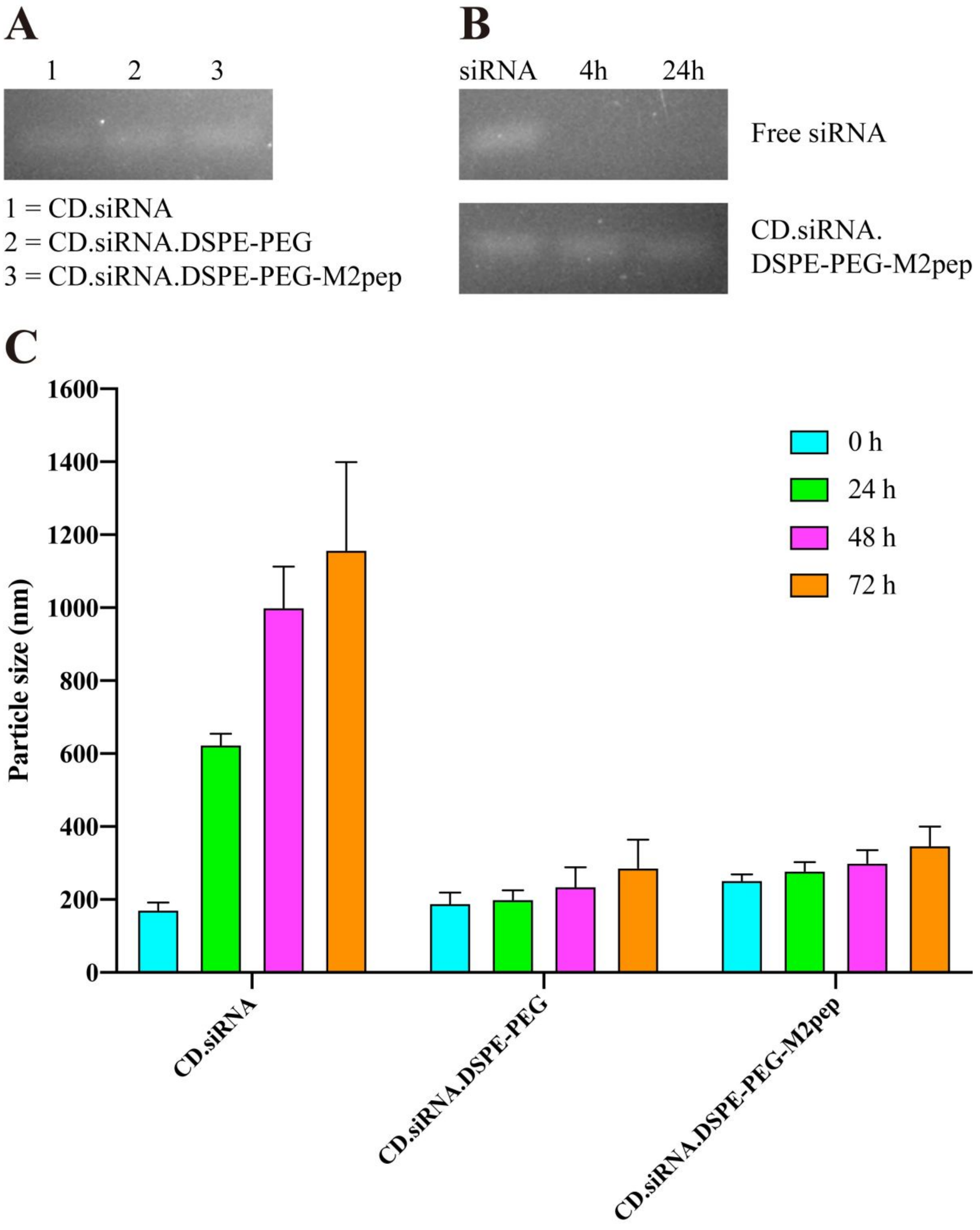

Figure S4. (A) CD.siRNA, CD.siRNA.DSPE-PEG, and CD.siRNA.DSPE-PEG-M2 were treated with heparin (1000 I.U./mL) for 1 h at room temperature to release siRNA. siRNA was confirmed by using 1% agarose gel electrophoresis, the resulting gels were imaged under UV. (B) Stability of naked siRNA (0.5 μg) and siRNA formulated with CD.DSPE-PEG-M2 after incubation for 4 h and 24 h in 50% FBS at 37 ℃. After incubation, CD.siRNA.DSPE-PEG-M2 was treated with heparin (1000 I.U./mL) to release siRNA. siRNA was confirmed by using 1% agarose gel electrophoresis, the resulting gels were imaged by UV. (C) Stability of CD.siRNA, CD.siRNA.DSPE-PEG, and CD.siRNA.DSPE-PEG-M2 in 90% Opti-MEM medium for 24 h, 48 h and 72 h (n=6, mean ± SD). Particle sizes were measured by DLS.

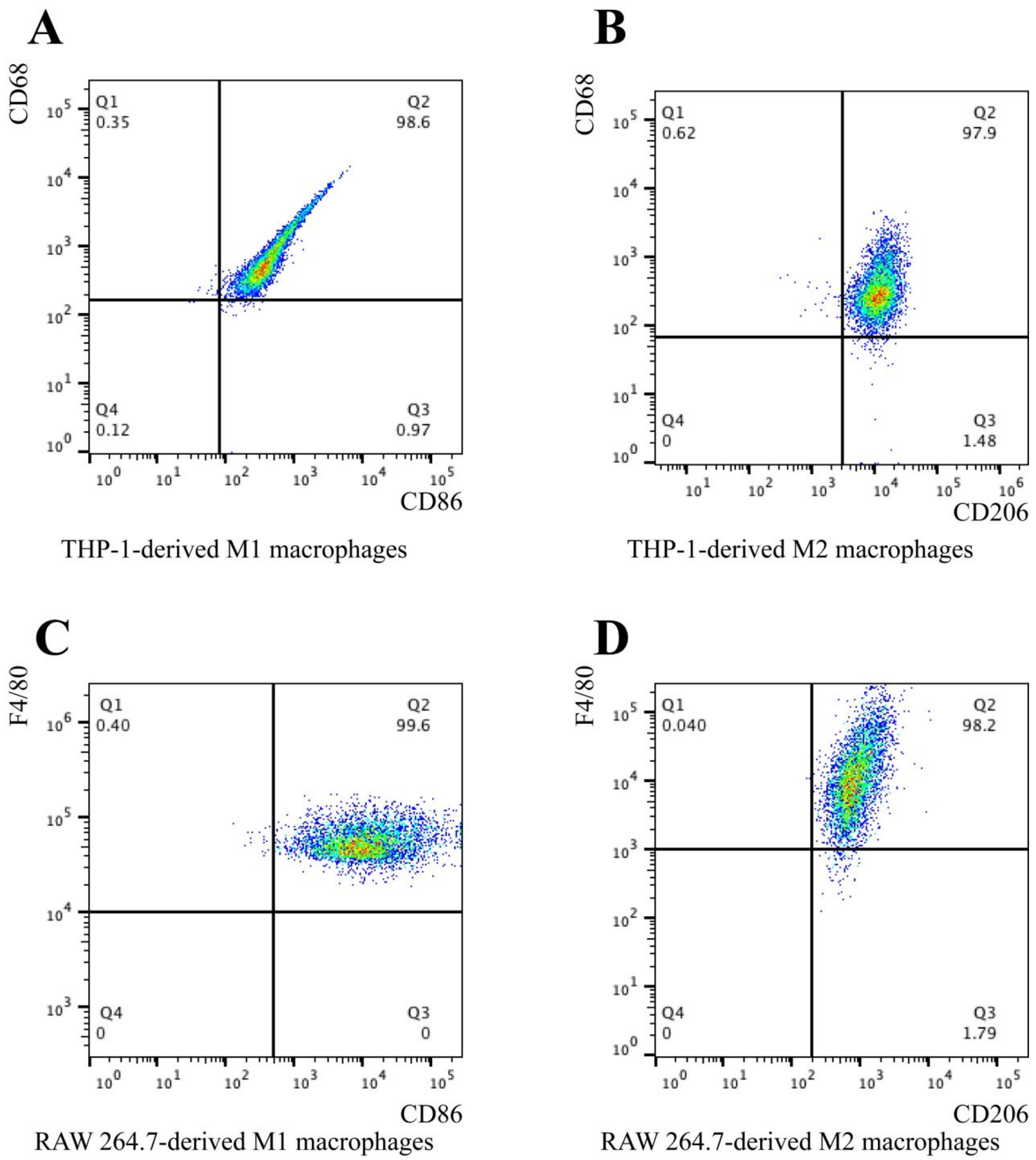

Figure S5. The expression of human macrophages marker (CD68) and M1/M2 macrophage markers (CD86/CD206) on THP-1-derived M1 (A) or M2 macrophages (B); and the expression of mouse macrophages marker (F4/80) and M1/M2 macrophage markers on RAW 264.7-derived M1 (C) or M2 macrophages (D), as measured by FACS.

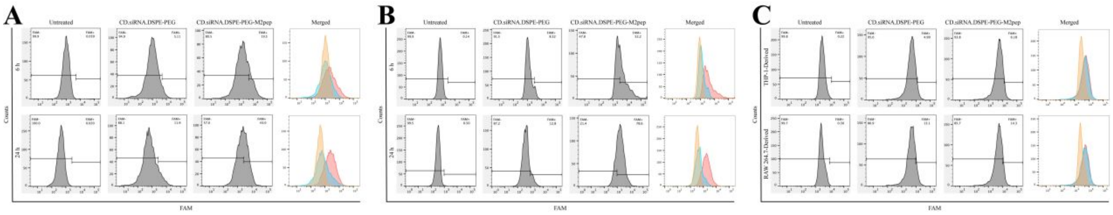

Figure S6. Representative flow cytometry figures of cellular uptake of CD NPs on (A) THP-1- and (B) RAW 264.7-derived M2 macrophages after 6 h or 24 h transfection, and (C) competitive uptake study.

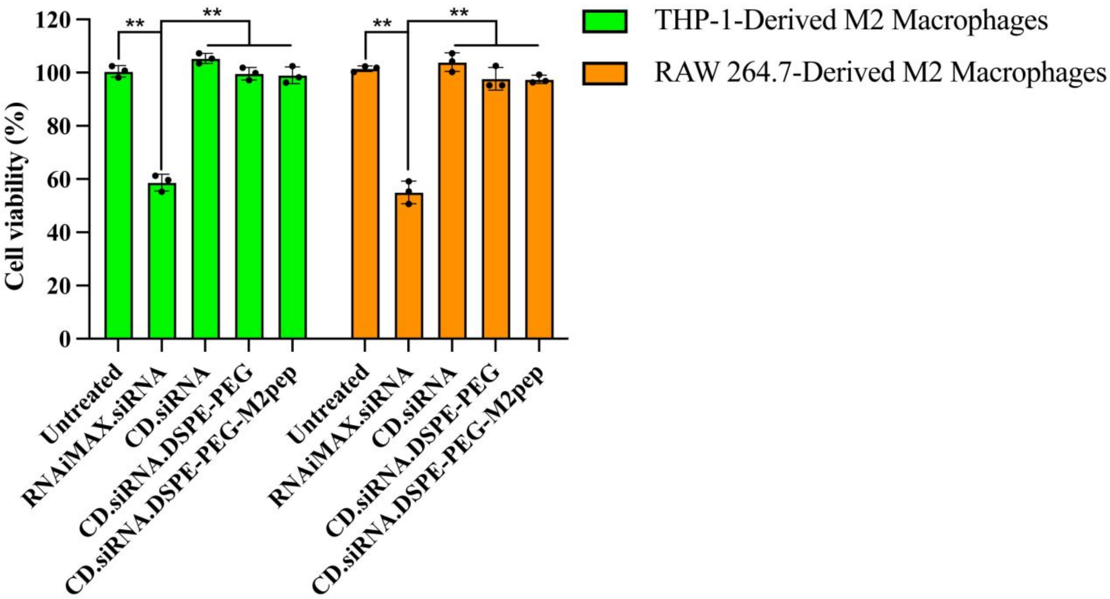

Figure S7. THP-1- or RAW 264.7-Derived M2 macrophages were treated with different CD formulations and positive control RNAiMAX for 24 hrs. Then CCK-8 was added with cells to measure cell viability (n = 3, mean ± SD,** $p < 0.01$).

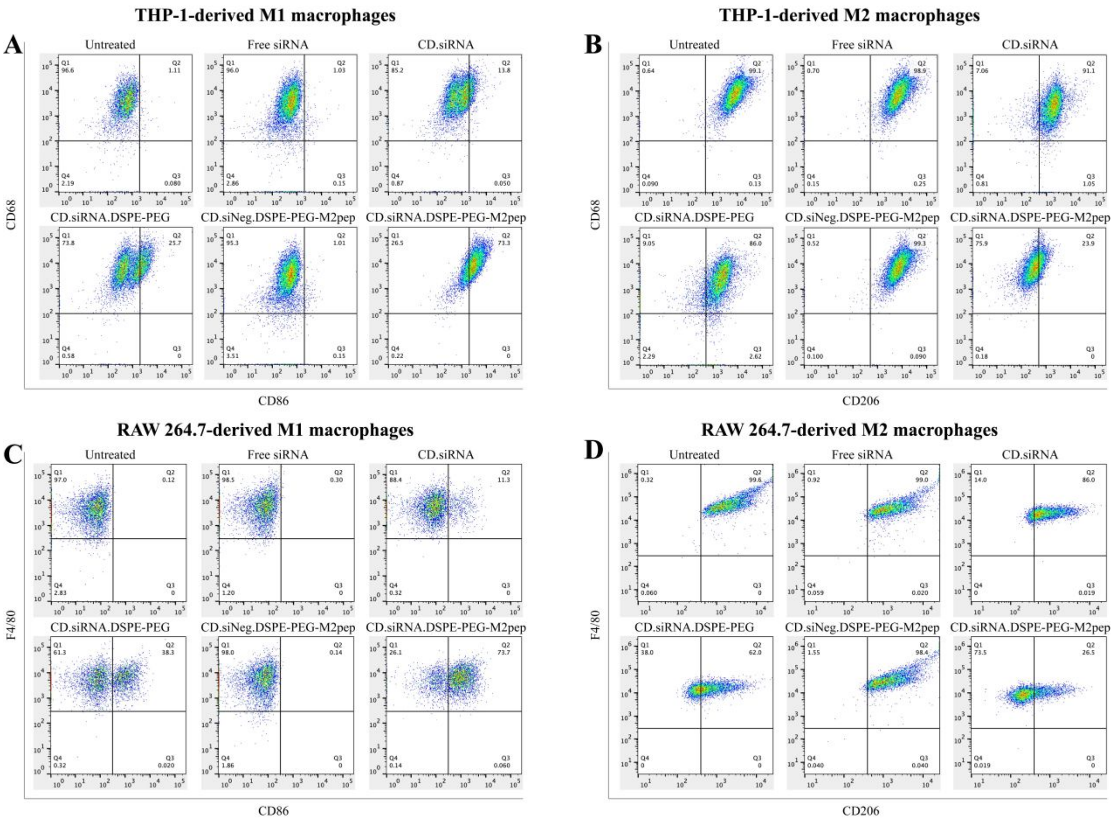

Figure S8. Representative flow cytometry figures of cellular uptake of the expression of the human macrophage marker CD68 and M1/M2 marker (A) CD86/ (B) CD206 on THP-1-derived M2 macrophages, and the mouse marker F4/80 and M1/M2 marker (C) CD86/ (D) CD206 on RAW 264.7-derived M2 macrophages after transfection.

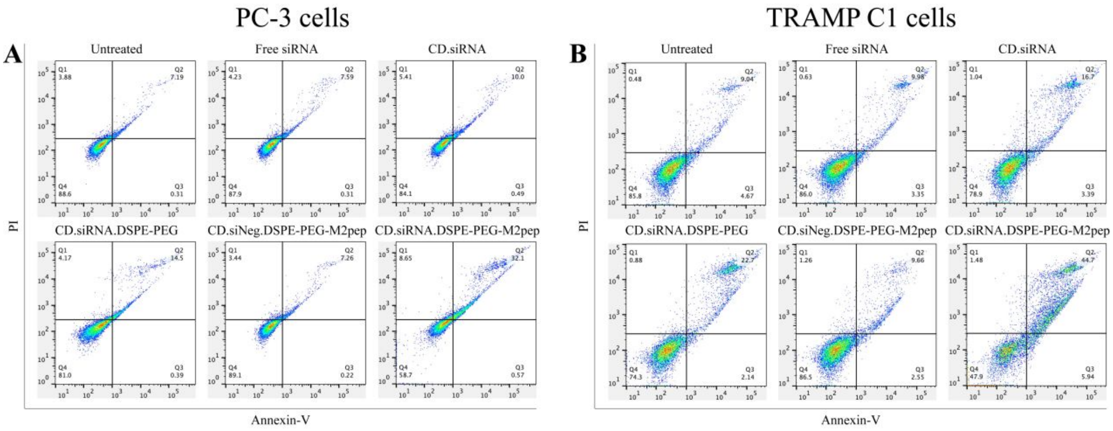

Figure S9. Representative flow cytometry figures of (A) PC-3 and (B) TRAMP C1 cells apoptosis.

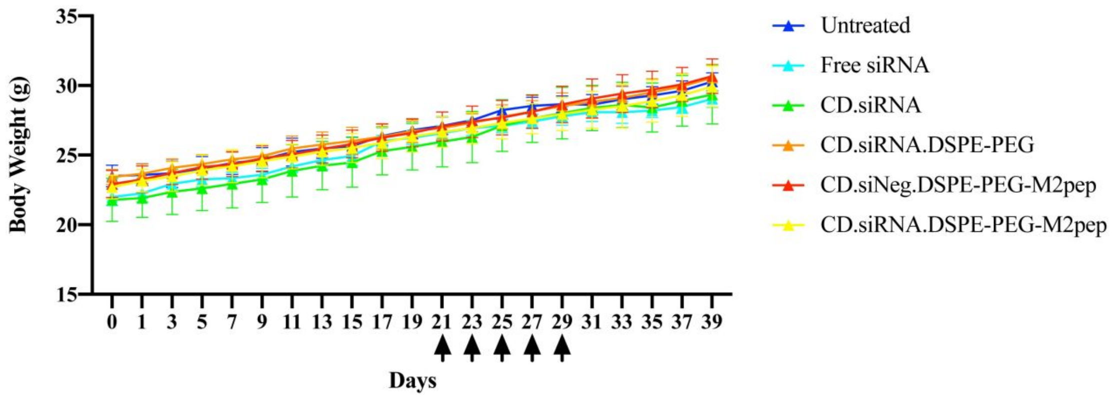

Figure S10. Body weight changes of prostate cancer-bearing models during anti-tumour effects study (n = 5, mean ± SD).

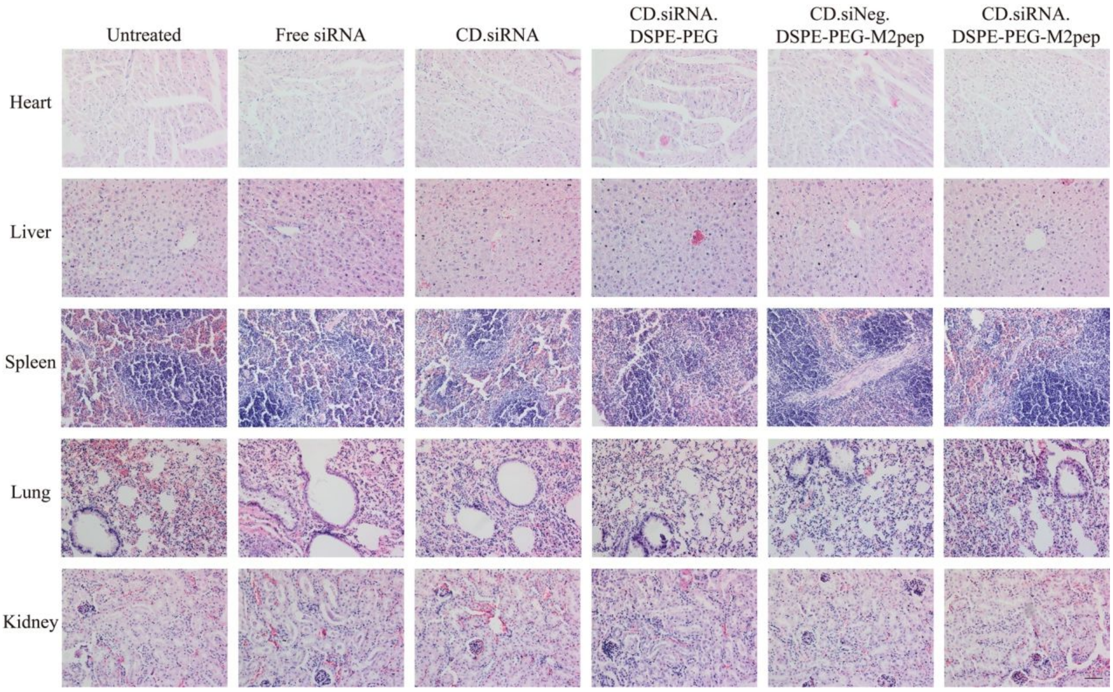

Figure S11. H&E staining of major organs sections among 6 groups. Scale bar = 50 μm.
